# Mask- and Coverage-Aware Traffic-State Assessment Under Sparse Probe-Vehicle Sensing

**DOI:** 10.3390/s26196280

**Published:** 2026-10-03

**Authors:** Dingxin Wu, Zheng Xu, Zhiyuan Wang, Kai Huang, Hong Ki An, Dewen Kong, Hongxin Wang, Zhicheng Shen

**Affiliations:** 1 Faculty of Transportation Engineering, Huai’an University, Huai’an 223001, China; 212503610605@hau.edu.cn; 2 Faculty of Computer & Software Engineering, Huai’an University, Huai’an 223001, China; 212409611261@hau.edu.cn; 3 State Key Laboratory of Comprehensive PNT Network and Equipment Technology, School of Instrument Science and Engineering, Southeast University, Nanjing 211189, China; kaihuang@seu.edu.cn; 4 School of Civil Engineering, Universiti Sains Malaysia, Engineering Campus, Nibong Tebal 14300, Pulau Pinang, Malaysia; anhongki77@usm.my; 5 Beijing Key Laboratory of Traffic Engineering, Beijing University of Technology, Beijing 100124, China; kongdw@bjut.edu.cn; 6 School of Transportation Engineering, East China Jiaotong University, Nanchang 330013, China; 2026138082303006@ecjtu.edu.cn (H.W.); 2026138082303007@ecjtu.edu.cn (Z.S.)

**Keywords:** probe-vehicle sensing, sparse traffic observations, observation mask, coverage-aware assessment, traffic-state reliability, large language models

## Abstract

**Highlights:**

**What are the main findings?**

- Sparse probe-vehicle coverage changes both prediction support and the reliability of an operational traffic-state severity alert; it cannot be treated as a preprocessing detail.
- Among deterministic variants, speed plus mask provided the strongest overall discrimination; a balanced RBF-SVM had higher mean grouped out-of-fold accuracy, but no method was uniformly superior across headline and false-severe metrics.

**What are the implications of the main findings?**

- Traffic monitoring systems should expose speed, observation support, and uncertainty together, and route weakly supported traffic-state alerts to conservative handling or review.
- Across the nine controlled-missingness conditions, evaluable target support varied systematically with mechanism and severity; MAE and RMSE must therefore be interpreted together with observed-target support.

**Abstract:**

Probe-vehicle sensing can extend traffic observation beyond fixed detectors, but its sparse and uneven coverage complicates the interpretation of predicted traffic states. This study develops a mask- and coverage-aware framework for regional traffic-state assessment from sparse probe-vehicle speed observations. Segment-level data from Xuzhou, China, were aggregated into 32 regions at 15 min resolution; only 0.157% of segment–time cells were observed. A regional target was observable whenever at least one segment in its region was observed, and the observed-target ratio across the controlled test conditions ranged from 12.1% to 23.9%. Nine controlled-missingness conditions were used to evaluate one-step speed prediction, followed by deterministic evidence-fusion assessment using predicted speed, observation masks, coverage history, and neighboring-region evidence. Reference labels for 130 stratified region–time cases were independently assigned and adjudicated by two annotators. Among the evaluated deterministic variants on this cohort, the speed-plus-mask rule achieved the strongest overall classification performance, with 69.2% accuracy and 63.7% Macro-F1. Because 69.2% equals the majority-class share of the cohort by construction (90 of 130 cases are low severity), the rule’s advantage is its cross-class discrimination (Macro-F1) rather than overall accuracy alone. Adding coverage reduced the false-severe rate from 5.6% to 2.8%, but also reduced overall classification performance, demonstrating an over-escalation-control–classification trade-off. On a limited 16-case protocol, a fixed, schema-constrained large language model achieved 97.5% modal-label repeatability across five repetitions but only 32.0% Macro-F1; its limited and imbalanced support precludes any full-cohort effectiveness claim. The findings show that sensing support should be retained as traffic-state assessment evidence and reported alongside prediction and traffic-state alert performance. On the same 130 cases, a balanced RBF-SVM reached 75.0% mean grouped out-of-fold accuracy and 64.6% Macro-F1, but all four headline-metric paired intervals versus speed plus mask crossed zero and its false-severe rate was higher (9.7% versus 5.6%); no method was uniformly superior.

## 1. Introduction

Regional traffic-state monitoring supports congestion response, traveler information, route guidance, and day-to-day traffic management. These functions depend on timely information about speeds and their changes across a road network. Fixed detectors can provide regular measurements at installed locations, but their deployment and maintenance constrain spatial coverage. By contrast, global positioning system (GPS)-enabled fleets, connected vehicles, and other mobile probes offer an increasingly important source of network-wide observations for public agencies and traffic operators [1,2,3]. The practical advantage of probe-vehicle sensing (PVS) is its spatial flexibility: the same fleet can contribute observations across many links without requiring a dedicated detector at every location.

This flexibility also creates a measurement problem. A road segment is observed only when one or more probes traverse it in a time window; consequently, the observation process depends on fleet penetration, route choice, traffic demand, road class, time of day, and local accessibility. The resulting segment–time speed matrix is sparse and uneven rather than a regularly sampled detector panel [2,4]. In this setting, a low reported or predicted speed and a low level of observation support have different meanings. The former may be evidence of a traffic deterioration, whereas the latter primarily limits the reliability with which a deterioration can be inferred. Treating both as interchangeable signals can convert a sensing outage or a weakly supported extrapolation into a high-severity traffic-state alert.

Traffic forecasting research has made substantial progress in modeling spatial and temporal dependence. Graph-based and recurrent architectures, including diffusion convolutional recurrent neural networks (DCRNNs), spatiotemporal graph convolutional networks (STGCNs), Graph WaveNet (GWNet), and adaptive graph convolutional recurrent networks (AGCRNs), are representative examples of models designed for networked traffic series [5,6,7,8]. Recent reviews show that graph construction, spatial representation, and temporal fusion remain central design choices for traffic forecasting [9]. However, a low prediction error alone does not answer the operational question faced by a monitoring service: whether the available speed, temporal history, and neighboring evidence are sufficient to justify an ordinal operational traffic-state severity alert. Related work on incomplete traffic data has largely concentrated on reconstructing unobserved values. Statistical, tensor-based, and deep learning approaches seek to improve the completeness of a traffic matrix under specified missing patterns [4,10]. This objective is valuable, but it differs from reliability-aware assessment. Imputation seeks a plausible value for an unavailable cell; assessment must decide whether the combination of a predicted value and its sensing support warrants a low-, medium-, or high traffic-state severity interpretation. If coverage and observation masks are hidden during assessment, an apparently severe speed drop can be over-interpreted even when the observation process is too weak to support a confident alert.

Large language models (LLMs) have recently been studied for intelligent transportation systems (ITSs), including traffic analysis, control, explanation, and multimodal decision support [11,12,13]. These studies motivate the use of language models as structured reasoning or communication components, but they also identify concerns about numerical reliability, latency, deployment cost, and the consistency of reasoning with a target task [11,12]. For sparse traffic-state assessment, an LLM should therefore not be treated as an unverified replacement for observed evidence or human judgment. Its role must be bounded by fixed inputs, schema-constrained outputs, and a repeatability audit.

Taken together, the literature establishes the value of probe sensing, missing-data modeling, spatiotemporal prediction, and emerging LLM-based ITS assistance. It leaves a methodological gap at their intersection. Existing studies rarely evaluate whether a predicted regional speed is sufficiently reliable for assessment after accounting jointly for the observation mask, current and historical coverage, temporal changes, neighboring-region evidence, and a human-grounded reference label. They also seldom connect a controlled-missingness prediction comparison to an explicit operational policy for cases whose sensing support is too weak for routine automatic handling, despite the broader relevance of uncertainty-aware prediction and selective prediction to severity-classification–coverage trade-offs [14,15,16]. Moreover, LLM-based traffic studies generally evaluate task outputs or simulated control actions rather than separating protocol stability from assessment effectiveness on sparse, real-world evidence. This study addresses these gaps by treating sensing support as a first-class assessment input rather than as a preprocessing detail.

Recent studies have extended sparse-trajectory research in four complementary directions: supervised reconstruction of sparse license-plate-recognition trajectories [17], GPS-based road-segment congestion estimation with dynamic critical bottlenecks [18], trajectory datasets spanning multiple traffic states and state transitions [19], and fleet-data-based traffic modeling [20]. These lines of work primarily target path reconstruction, congestion indexing, transition characterization, or network-level traffic modeling; whereas the present study targets the reliability of a coverage-aware ordinal regional assessment against adjudicated human labels.

Sparse support is also a design variable in adjacent vehicle-state-estimation research. Zhu et al. [21] combine sparsity-aware sensor selection with privacy-preserving zonotopic fusion filtering for cloud-based vehicle tracking, explicitly trading communication support against estimation and privacy objectives. In a complementary multi-vehicle setting, Zhu et al. [22] use distributed set-membership state estimation and sparse topology optimization to improve situational awareness under unknown inputs and bounded noise. These studies concern vehicle-state estimation and sensing architecture rather than regional traffic-state classification, but they reinforce the need to keep sensing support and estimation uncertainty visible rather than treating missing observations as neutral values.

The principal contributions of this study are threefold. First, we execute an auditable mask- and coverage-aware evaluation design for regional traffic-state assessment under sparse PVS. The design treats sensing support as a first-class input, retains the observation mask, current and historical coverage, zero-coverage history, and neighboring evidence through prediction and assessment, and evaluates prospective outputs against a frozen 130-case adjudicated cohort. The information boundary is explicit: target-observation status and target-time speed are available only to retrospective human reference labeling, not to the prospective components. Second, we show that prediction error cannot be interpreted independently of evaluable sensing support. Across nine controlled-missingness conditions, the observed-target set changes systematically with mechanism and severity; at high severity, temporal-block, random, and segment-index-block conditions retain 6719 (12.1%), 10,860 (19.6%), and 11,343 (20.5%) targets, respectively. MAE and RMSE are therefore reported jointly with observed-target support rather than treated as standalone evidence of sensing robustness. Third, we provide a same-cohort, human-grounded assessment comparison covering five fixed deterministic variants, a training-fold majority baseline, class-balanced multinomial logistic regression, and a class-balanced RBF-SVM. The learned comparators use repeated grouped out-of-fold evaluation with fold-local preprocessing, and paired cluster-bootstrap inference is reported alongside class-sensitive metrics. We additionally audit a prespecified 243-configuration threshold grid without replacing the primary rule, quantify a coverage-selective manual-review policy, and test ranking sensitivity after excluding disputed or lower-confidence labels. Separately, we audit a fixed, schema-constrained LLM assistance protocol on 16 cases across repeated calls. This limited analysis measures schema validity and protocol repeatability, not full-cohort assessment effectiveness, and is not presented as a principal methodological contribution or as a replacement for deterministic or human-grounded evaluation.

The remainder of this paper is organized as follows. Section 2 reviews sparse probe-vehicle sensing, missing-data-aware traffic modeling, reliability-aware assessment, and LLM-based ITS assistance. Section 3 describes the study data, controlled-missingness protocol, prediction and assessment methods, annotation procedure, and evaluation design. Section 4 reports the empirical results, including matched prediction baselines, threshold robustness, and coverage-selective assessment. Section 5 discusses operational implications, failure modes, and limitations. Section 6 concludes the paper.

## 2. Related Work

### 2.1. Probe-Vehicle Sensing and Sparse Traffic-State Estimation

Probe-vehicle sensing uses moving vehicles as opportunistic observation units. Compared with a fixed-detector network, it can expand the spatial footprint of traffic observation and can support monitoring where permanent infrastructure is unavailable or expensive. Public-agency practice has consequently incorporated vehicle probe and cellular GPS products into traffic monitoring and incident communication [1]. Research has also shown that GPS speed measurements can contribute to motorway state estimation under limited sensing resources [2], while advanced driver-assistance-system probe trajectories can be used to estimate traffic density when the sampling rate and the estimation scale are carefully considered [3].

The defining characteristic of this sensing paradigm is that observations are generated by vehicle movement rather than by a continuously installed sensor. A segment can be unobserved because no probe used it, because the fleet is small, or because the time window is short; none of these conditions directly implies free flow, congestion, or a sensor fault. Sparse observation is therefore a property of the sensing mechanism, not simply independent random noise. This distinction is important for the present study: we aggregate segment observations to regional speeds to create an operational monitoring unit, but we retain regional coverage and zero-coverage history rather than assuming aggregation removes uncertainty.

Prior PVS studies primarily aim to estimate a traffic variable, reconstruct a field, or quantify the sampling needed for a target estimation accuracy. Recent work spans deep-learning traffic prediction reviews, comprehensive speed-prediction analyses, and heterogeneous-sensor fusion [23,24,25]. Sparse-mobile-crowdsensing transformers, sparse movement-speed factorization, sparse road-network prediction, GraphSAGE-based segment forecasting, and varying-speed-level experiments further demonstrate that sparse network observations remain a substantive modeling setting rather than a peripheral data-cleaning issue [26,27,28,29,30]. These are necessary foundations, but they do not by themselves define how an operator should interpret a low-speed prediction when the evidence supporting it is sparse or conflicting. Our focus is thus downstream of state estimation: reliability-aware ordinal assessment using the available sensing evidence.

Fleet-data studies provide the closest network-level context for this sensing setting. Tettamanti et al. [20] use fleet observations for network traffic modeling, while Feng et al. [19] curate expressway trajectories covering multiple traffic states and their transitions. These works demonstrate how mobile trajectories can characterize network dynamics and state changes; our framework instead retains regional observation support as an explicit assessment input and evaluates the resulting three-level decision rather than treating traffic modeling or dataset construction as the endpoint.

### 2.2. Missing-Data Modeling and Mask-Aware Traffic Forecasting

Traffic missing-data research covers a broad family of approaches, including temporal interpolation, neighborhood methods, matrix and tensor completion, and deep spatiotemporal imputation. Traffic-forecasting surveys have documented the importance of graph construction and spatial–temporal learning choices [31,32], while recent missing-data surveys and methods emphasize that missingness mechanisms, missing rates, temporal dependence, spatial information, and evaluation assumptions materially affect imputation results [4,33]. Uncertainty-aware prediction, graph-based completion, missing-information imputation, and mask-aware graph models explicitly seek to represent incomplete traffic evidence rather than relying only on zero pre-filling [10,14,34,35]. Forecasting models likewise use increasingly rich spatial and temporal mechanisms. DCRNN models network propagation through diffusion processes [5]; STGCN combines temporal convolutions and graph convolutions [6]; GWNet learns adaptive spatial dependencies with dilated temporal convolutions [7]; and AGCRN learns node-adaptive graph structure and recurrent parameters [8]. Subsequent work has introduced comprehensive forecasting benchmarks, spatial–temporal fusion graphs, graph ordinary differential equations, decoupled dynamic graphs, propagation-delay-aware transformers, and adaptive-embedding transformers [36,37,38,39,40,41]. Real-time joint prediction/imputation and tensor approaches also illustrate the close relationship between reconstruction and traffic forecasting [42,43]. Such work establishes that masks and spatial structure can be useful predictive inputs, but its main outcome is generally point prediction accuracy.

The present study makes two distinctions. First, reconstructed values are not treated as direct observed ground truth for the assessment reference set. Second, a prediction model may use coverage information, but an assessment module must still expose whether a severe score is supported by current and historical observations. We therefore evaluate controlled random, temporal-block, and segment-index-block missingness separately, preserve a target observation mask in prediction metrics, and compare assessment-rule variants that selectively incorporate temporal, coverage, and spatial evidence. This design does not claim to outperform specialized imputation methods; it asks how missingness affects the reliability of an operational traffic-state decision.

Li et al. [17] address sparse observations by learning to reconstruct trajectories from sparse license-plate-recognition records. That objective is complementary but distinct: trajectory reconstruction seeks plausible unobserved paths, whereas our evaluation does not convert reconstructed paths into reference evidence; it preserves the mask and coverage variables and measures how missingness affects both speed prediction and downstream assessment.

The choice of benchmark is also consequential under sparse support. A complex spatiotemporal model can appear favorable when it is compared only with a persistence or historical-average reference, while a feature-based model may exploit recent speed, coverage, neighborhood summaries, and time-of-day information effectively. Tree-based methods also remain competitive baselines for many medium-sized tabular prediction problems [44]. Conversely, aggregate MAE can conceal that different missingness mechanisms leave different subsets of targets evaluable. General forecasting-benchmark work likewise emphasizes the importance of comparable evaluation protocols [36]. For this reason, a reliable comparison should hold the prediction target mask and split fixed across models, report all mechanisms and severities rather than only a pooled score, and distinguish a prediction benchmark result from an operational traffic-state severity alerting claim. The present work follows this logic by comparing the masked recurrent model with historical average, persistence, moving average, ridge regression, and HistGB under the same nine datasets and test masks.

### 2.3. Traffic Forecasting, Alert Reliability, and Human-Grounded Assessment

Short-horizon traffic prediction is often evaluated with mean absolute error (MAE), root mean squared error (RMSE), or related aggregate measures. These quantities are essential for assessing a forecast, but they do not express the asymmetric operational cost of a false-severe traffic-state alert. A high-severity traffic-state alert can trigger attention, intervention, or public communication; under sparse sensing, avoiding unsupported escalation may therefore be as important as maximizing aggregate classification accuracy. The probe-data literature and ITS practice both motivate using measurement availability as part of the interpretation of a traffic-state estimate [1,2]. Reliable alerting requires a reference standard that is separated from the system being evaluated. In this study, two annotators independently reviewed region–time case records containing speed history, coverage information, prediction outputs, and neighbor summaries, while deterministic-rule and LLM outputs were withheld. The disagreement cases were then adjudicated. This design does not claim that human labels are an infallible ground truth; instead, it provides a transparent, human-grounded reference for comparing deterministic assessment-rule variants and for reporting their limitations through Macro-F1, false-severe rate, and coverage-stratified results. Because annotators saw the target-time truth, the reference labels are retrospective human-grounded labels created with access to target-time evidence; they are not generated under the same information set available to the prospective assessment components.

Accordingly, our contribution is not a new traffic-state severity threshold asserted to be universally optimal. It is an evaluation design in which a traffic-state severity rule is judged by both its ordinal agreement with adjudicated labels and its tendency to over-escalate under sparse support. This distinction is central to interpreting the coverage-aware rule: reducing over-escalations can be operationally desirable even when it lowers discrimination in the lowest-coverage stratum.

At the traffic-state level, Xu et al. [18] estimate urban road-segment congestion from GPS trajectories while accounting for dynamic critical bottlenecks, and Feng et al. [19] provide trajectories explicitly organized around multiple traffic states and transitions. These studies motivate state-aware interpretation of trajectory data, but their outputs differ from the present target: a coverage-aware three-level regional assessment evaluated against independently annotated and adjudicated region–time cases.

Threshold and review-policy evaluation require further separation between description and optimization. A grid of transparent rule configurations can reveal whether a conclusion is stable to plausible design choices, but it must not be used to retrospectively select a better-performing threshold on the same adjudicated cohort and present it as a primary result. Recent selective-prediction research formalizes the related idea of trading prediction severity against the fraction of instances retained for automatic prediction [15,16]. Similarly, coverage can be used to rank cases for manual verification yet retained-case performance is conditional on the cases not routed to review and is not equivalent to end-to-end deployment effectiveness. We therefore report the full prespecified threshold grid without replacing the primary configuration and report fixed review fractions as an operational trade-off rather than a recommended universal cutoff.

### 2.4. LLMs for Intelligent Transportation and Structured Assessment Assistance

LLM research in transportation has expanded rapidly across traffic, safety, autonomous driving, and decision-support applications. A recent review of LLMs for ITS identifies more than 130 studies and emphasizes both the diversity of traffic-related applications and the need to address reliability, data, and deployment challenges [11]. Broader work on large models for ITS similarly highlights task alignment, computational cost, latency, and practical deployment as unresolved concerns [12]. LLM-based traffic signal-control studies illustrate the potential for language-mediated reasoning over structured traffic conditions, but they are predominantly evaluated in simulation or control settings [13].

Multi-agent conversational frameworks provide a general implementation pattern for assigning bounded subtasks to distinct roles, but role decomposition alone does not validate factual or assessment correctness [45]. Moreover, controlled evidence on intrinsic LLM self-correction indicates that unsupported revision can fail to improve reasoning and can sometimes degrade it [46]. These findings support a limited role for an LLM in sparse traffic-state assessment: it can organize explicitly provided evidence into a schema-valid explanation and identify missing or conflicting evidence for review. They do not justify allowing an LLM to invent incidents, weather, or unobserved traffic facts, nor do they justify treating a fluent explanation as proof of assessment accuracy. For this reason, our protocol supplies only structured case evidence, excludes the target true speed and target observation outcome, fixes prompts and temperature, validates every JSON output, and reports repeated-call modal-label stability separately from classification effectiveness.

### 2.5. Summary of Research Gaps and Positioning

The literature reviewed above reveals three principal gaps that motivate this study.

**Gap 1: Sparse probe sensing is often treated as a reconstruction problem rather than as traffic-state assessment evidence.** Probe observations are naturally intermittent because vehicles act as opportunistic moving sensors. Imputation and mask-aware prediction studies appropriately seek to recover or forecast unavailable traffic values [4,10,33]; however, fewer studies retain current coverage, zero-coverage history, and target observability as evidence that constrains the interpretation of an operational traffic-state severity alert. A low predicted speed under weak sensing support should not be interpreted in the same way as a low speed corroborated by sustained observations.

**Gap 2: Forecasting accuracy and assessment reliability are commonly evaluated as separate questions.** Graph and Transformer predictors provide increasingly capable spatiotemporal modeling tools [39,40,41], but their usual MAE/RMSE evaluation does not quantify whether an ordinal high-severity traffic-state alert is supported by the available measurements. Under sparse PVS, an assessment comparison requires common human-grounded cases and metrics that expose over-escalation, especially the false-severe rate, in addition to aggregate accuracy. This is a decision-reliability requirement rather than a claim that a single threshold is universally optimal.

**Gap 3: Sparse-sensing studies rarely connect matched model comparisons, selective review, and bounded decision-support protocols within one auditable evaluation design.** Prediction studies commonly report aggregate error for one or several baselines, but do not always hold target support visible across methods and missingness mechanisms. General forecasting benchmarks and selective-prediction studies, respectively, motivate comparable evaluation protocols and explicit severity-classification–coverage trade-offs [15,16,36]. Decision-support studies similarly rarely quantify what changes when the weakest-support cases are withheld for verification. Within this broader governance problem, LLM-based transportation assistance also requires fixed evidence restrictions and a protocol-stability audit that is reported separately from assessment effectiveness; fluent or repeatable outputs do not by themselves establish numeric validity [11,12,13]. An operationally relevant study should therefore report matched model comparisons on common masks, the conditional trade-off created when low-coverage cases are routed to review, and any auxiliary LLM evidence as a bounded protocol audit. These analyses should remain descriptive unless independently validated as a deployed human–machine workflow. To address these gaps, this paper develops a mask- and coverage-aware traffic-state assessment framework for sparse PVS. It keeps prediction, observation support, deterministic evidence fusion, human adjudication, and the bounded LLM protocol as linked but separately auditable stages. The analysis then evaluates controlled missingness, matched non-neural prediction baselines, cohort-level assessment performance, coverage-stratified false-severe behavior, threshold robustness without retuning, a coverage-selective review analysis, a representative evidence trace, and limited-sample LLM stability. In this design, observation support is carried from sensing through prediction, assessment, and selective review as a first-class quantity rather than being discarded after preprocessing. Thus, the central contribution is not another universal forecasting architecture; it is a transparent evaluation design for deciding when a predicted traffic signal can support a traffic-state severity alert.

Table 1 summarizes the resulting research positioning. Prediction remains necessary, but the principal output is an auditable traffic-state-severity interpretation whose uncertainty is tied to sensing support. The LLM component is evaluated for fixed-protocol stability on a limited support and is not used to create the human reference labels.

## 3. Materials and Methods

### 3.1. Problem Setting, Terminology, and Data Representation

We studied traffic-state assessment from sparse probe-vehicle sensing (PVS) in Xuzhou, China. Throughout this paper, the ordinal labels low, medium, and high denote traffic-state severity or operational attention levels inferred from speed, observation support, and neighboring evidence. They do not represent road-safety risk, crash probability, conflict risk, injury severity, or any other independently measured or validated traffic-risk outcome. The source data cover 1 January to 31 March 2026 at a 15 min resolution, yielding 8640 temporal windows. The raw records were first restricted to moving observations within the study bounding box. Coordinates were converted from GCJ-02 to WGS84 and map matched to directed OpenStreetMap road segments using an 80 m maximum match distance. Of 750,382 eligible records, 685,805 (91.4%) were matched; records without a valid segment or with speeds outside 0–140 km/h were excluded before aggregation. Matched speeds were averaged within each segment–15 min cell, with the number of matched records retained as the cell sample count.

The 685,805 successfully map-matched records included one record with a speed of 143 km/h. After applying the prespecified 0–140 km/h validity filter, 685,804 valid matched records contributed to the segment–time and region–time aggregation matrices.

The analysis segment universe contains the 49,645 segments with at least one valid matched observation during the three-month study period, rather than every segment in the larger OpenStreetMap extract. Thus, the denominator of regional coverage is the number of historically observable analysis segments assigned to that region; it does not include roads that were never observed anywhere in the study period. Coverage is therefore a within-region sensing-quality measure rather than a probe count: regions with more observable analysis segments require more observed segments to reach the same coverage value, and the denominator is fixed across the study period. The resulting raw speed matrix is nevertheless extremely sparse: only 674,600 of 428,932,800 segment–time cells were observed (0.157%). To obtain an operational assessment unit while preserving observation support, these analysis segments were aggregated into 32 regions.

The regional partition was fixed before prediction and assessment. The partition was constructed from the complete three-month record (segment daily profiles, NMF embedding, and K-means clustering), so it is a transductive, system-design step analogous to an infrequently reconfigured monitoring network rather than a train-only grouping; partition stability under a train-only construction window remains to be evaluated (Section 5.6). For each segment, a 96-slot daily speed profile was formed from sample-count-weighted observations, missing slots were filled with the corresponding cross-segment slot mean, and each filled profile was normalized by its segment mean for shape comparison. A 12-component non-negative matrix factorization (NMF) embedding was combined with standardized segment coordinates, and 32 initial clusters were produced by K-means. High-confidence cluster cores were then expanded and refined over the segment adjacency graph with a graph-snake objective that balances embedding fit and boundary continuity (spatial-feature weight 1.5; boundary/data/inertia weights 0.6/1.0/0.2). The fixed 32-region scale is an analysis design choice rather than a tuned optimum; the complete mapping, partition configuration, seed assignments, and energy log are retained in the reproducibility package.

The segment-level observability and region-level target-observation ratio answer different questions and should not be compared as if they used the same unit. A regional target is recorded as observed when at least one segment assigned to that region is observed in that 15 min window; multiple sparse segment observations can therefore complement one another within a region. The observation mask M has entry $M_{t,r}=1$ when at least one analysis segment assigned to region r is observed in window t and $M_{t,r}=0$ otherwise; it records target availability rather than sensing strength, whereas C quantifies the amount of regional support. There are 32 × 8640 = 276,480 possible region–time cells before controlled masking. Across the nine test conditions, 12.1–23.9% of region–time targets remained observable. This does not imply that the raw segment data are dense; it states the support available after the explicitly defined regional aggregation and controlled masking.

Before controlled masking, 155,071 of the 276,480 region–time cells contained at least one observed segment and 121,409 (43.9%) had zero coverage. Across all region–time cells, the observed-segment count had a median of 1 (IQR 0–3; P90 7; P95 10; maximum 45), while the count of valid matched records contributing to aggregation had a median of 1 (IQR 0–3; P90 7; P95 11; maximum 53). Conditional on positive support, the corresponding medians were 3 for both measures, with P95 values of 13 observed segments and 14 matched records. Table A4 reports the complete distribution. These are matched-record counts, not counts of unique vehicles.

Let $x_{t,s}$ and $n_{t,s}$ denote the observed speed (km/h) and probe-vehicle count for segment s in time window t, respectively. Let g(s) map a segment to its region. For region r, the regional speed $R_{t,r}$ was computed as the probe-count-weighted mean of all observed segment speeds in that region. Missing regional speeds were retained as missing rather than interpreted as zero. Regional coverage $C_{t,r}$ was defined as the proportion of the region’s road segments with at least one observation in that window. Thus, speed and sensing support are separate quantities: a low speed is a traffic signal, whereas low coverage is primarily a reliability signal. Table 2 defines the notation used throughout this study.

The regional speed matrix R therefore has entry $R_{t,r}$ equal to the probe-count-weighted mean of all observed analysis-segment speeds assigned to region r in window t, and the entry remains missing when no segment is observed. The coverage matrix C has entry $C_{t,r}$ equal to the number of observed analysis segments in region r and window t divided by the total number of historically observable analysis segments assigned to r; this denominator is fixed over the study period.

(1) $$R_{t,r}=\frac{\sum_{s:g\left(s\right)=r}n_{t,s}x_{t,s}}{\sum_{s:g\left(s\right)=r}n_{t,s}},\;\;\;\;\;\;\;C_{t,r}=\frac{1}{\left|S_{r}\right|}\sum_{s\in S_{r}}⊮\left[n_{t,s}>0\right],$$

At the segment level, the observation mask is $m_{t,s}=⊮\left[n_{t,s}>0\right]$. The regional target mask used for model training and evaluation is

(2) $$M_{t,r}=⊮\left[\sum_{s:g\left(s\right)=r}n_{t,s}>0\right]$$

which ensures that an unavailable target is neither imputed as a true speed nor included in an error calculation.

### 3.2. Region-Level Aggregation and End-to-End Pipeline

**Figure 1:**
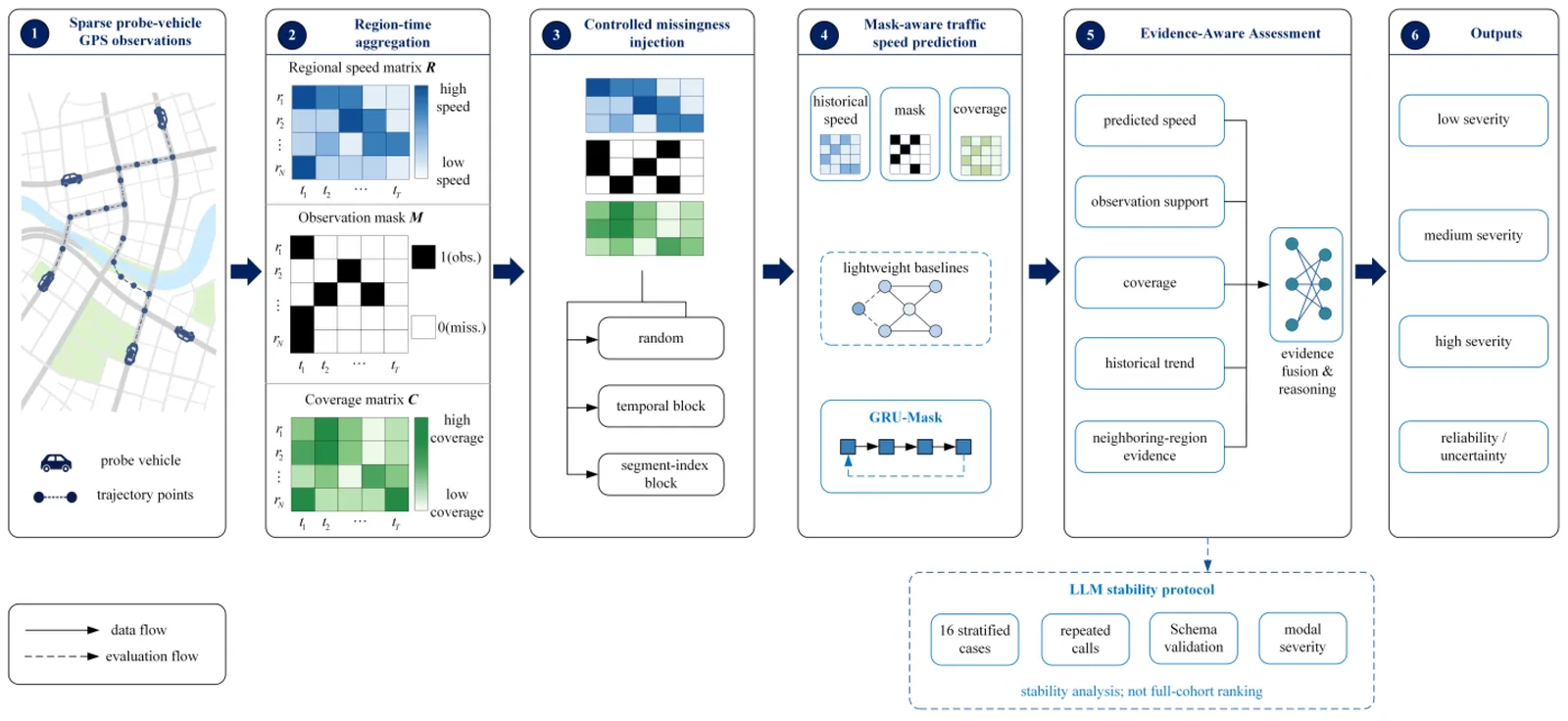
End-to-end architecture from sparse probe-vehicle observations to regional traffic-state assessment. Six modules are organized into four analytical stages: representation (modules 1–2), controlled missingness and prediction (modules 3–4), deterministic evidence-aware assessment and outputs (modules 5–6), and a separate dashed LLM stability branch. Speed, observation support, temporal history, and neighboring-region evidence remain distinct inputs. Adjudicated human labels and target-time truth are used only for offline reference evaluation and are not online inputs.

As summarized in Figure 1, the six diagrammed modules are organized into four analytical stages: sparse-observation preprocessing and region–time aggregation (modules 1–2; Section 3.1 and Section 3.2), controlled-missingness injection and mask-aware speed prediction (modules 3–4; Section 3.3 and Section 3.4), evidence-aware assessment (module 5; Section 3.5), and reported outputs with offline reference and stability evaluation (module 6 and the dashed LLM branch; Section 3.6 and Section 3.7). First, probe observations were aggregated into regional speed, coverage, and historical correlation features. Second, controlled missingness was injected into the sparse observations and region-level prediction datasets were constructed. Third, a GRU-Mask predictor estimated the next regional speed from a 12-window history. Finally, Figure 1 module 5 applies the five deterministic evidence-fusion variants described in Section 3.5 and sends their prospective severity outputs to module 6; the dashed LLM path is a separate 16-case stability audit and is not one of those deterministic variants. Human labels were collected and adjudicated independently of the traffic-state assessment-system outputs used for evaluation.

Accordingly, the Figure 1 construction chain is raw probe-vehicle GPS cleaning, coordinate conversion and OpenStreetMap segment matching, segment–15 min aggregation, fixed 32-region aggregation, construction of R, M, and C, and then controlled-missingness injection. At each region–time cell, R stores the probe-count-weighted observed speed (and remains missing when M=0), M records whether at least one analysis segment is observed, and C records the observed fraction of that region’s fixed set of historically observable analysis segments.

The output of the predictive stage was not treated as a complete measurement. Each assessment case retained the predicted speed, the latest observed speed, the speed drop from that observation, current and historical coverage, zero-coverage history, and summarized neighboring-region evidence. This representation makes it possible to distinguish a plausible traffic deterioration from an apparently severe signal generated under weak sensing support.

Temporal information sets and leakage controls. For an assessment target at t+1, the final input window is t and the preceding input history spans t−11 through t. The one-step prediction for the target region and every neighboring region is generated from this same historical information set. Table 3 distinguishes the information available to the prospective assessment components from the extra target-time evidence available only for offline human reference labeling. Consequently, neighboring-region evidence used by the deterministic rules and LLM consists of a neighbor’s latest input-window speed and coverage at t, its one-step prediction for t+1, and correlations stored in the frozen 130-case evidence package; it does not use an observed neighbor speed at t+1. The frozen package inherited a correlation matrix estimated over the complete three-month record, whereas missing-speed imputation used training-period means. For the revised implementation, regional correlations are estimated from the chronological 70% training partition. We additionally performed a post hoc sensitivity audit that replaced the 641 stored neighbor-correlation values with training-partition estimates while holding case identities, predictions, histories, neighbor identities and order, and adjudicated labels fixed. Because this audit does not reconstruct the original top-five-neighbor selection, it is interpreted as a robustness check rather than as a fully prospective replacement of the annotation evidence.

### 3.3. Controlled-Missingness Protocol

**Figure 2:**
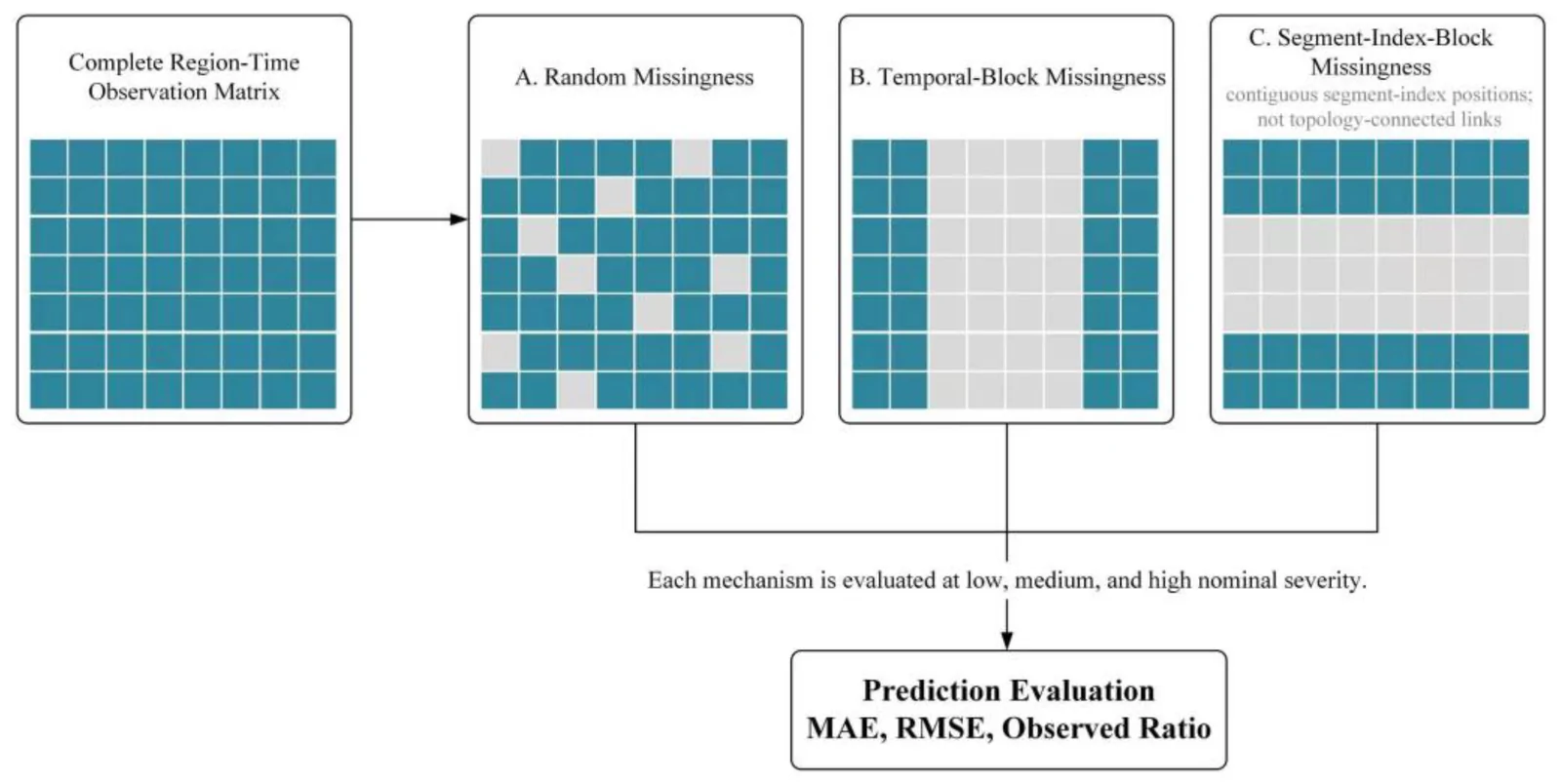
Controlled-missingness design. Random, temporal-block, and segment-index-block mechanisms were applied at three nominal severities while preserving the chronological data partition. Here, segment-index-block denotes a stress test that removes contiguous segment-index blocks; it does not imply topological or geographical contiguity. Teal cells indicate observed entries, whereas light-gray cells indicate masked or missing entries.

As shown in Figure 2, robustness beyond the naturally sparse observation pattern was tested with nine controlled-missingness conditions formed by crossing three mechanisms (random, temporal-block, and segment-index-block) with three nominal severities (10%, 30%, and 50%) within each chronological partition. The mechanisms were random missingness, temporal-block missingness, and segment-index-block missingness. Random missingness removes observed cells independently within each temporal split. Temporal-block missingness removes a contiguous interval of time windows within each split. Segment-index-block missingness removes observations from a contiguous block of segment indices; because segment indices follow the source data order rather than a verified spatial ordering of the road network, this mechanism is an index-based stress test rather than a demonstrated topology-aware outage. The nominal removal rates were 10%, 30%, and 50% for low, medium, and high severity, respectively.

In the retained frozen artifacts, this condition is stored under the legacy identifier spatial_block. The legacy identifier and the revised term refer to the same segment-index-block stress test; the archived identifier is retained solely to preserve traceability to the executed runs.

For a condition with binary removal indicator $d_{t,s}^{\left(a\right)}$, where a denotes the missingness mechanism and severity, the injected mask is

(3) $${\tilde{m}}_{t,s}^{\left(a\right)}=m_{t,s}\left(1-d_{t,s}^{\left(a\right)}\right),\;\;\;\;\;\;\rho^{\left(a\right)}=\frac{\sum_{t,s}m_{t,s}d_{t,s}^{\left(a\right)}}{\sum_{t,s}m_{t,s}}$$

Here, $\rho^{\left(a\right)}$ is the realized fraction of originally observed cells removed by the condition. The construction guarantees that artificial removal is applied only to cells observed in the original sparse matrix.

All masks were generated separately within the chronological training, validation, and test partitions. This prevents observations from one partition from influencing the missingness pattern in another and avoids leakage from the test period into model development. For every condition, we recorded the number of observed cells before and after removal, the realized density, the affected time windows, and the affected segments. Because the observed target set itself changes with missingness, prediction results are always interpreted jointly with the observed-target ratio.

### 3.4. Prediction Models and Training Protocol

For each missingness condition, a sample used the preceding 12 15 min windows (3 h) to predict the next window (h=1). The resulting dataset contained 8628 samples and 32 regional targets per sample. Samples were split chronologically into training, validation, and test sets with 6036, 863, and 1729 samples, respectively. Missing input values in R were filled with training-period regional means; target values were zero-filled only for storage and were excluded from the loss and all prediction metrics through the target observation mask M.

For target time t, the model input and target can be written as

(4) $$X_{t}=\left[\left(R_{t-11,:},C_{t-11,:}\right),\ldots ,\left(R_{t,:},C_{t,:}\right)\right],\;\;\;\;\;\;\;\;\;y_{t+1}=R_{t+1,:}$$

where missing values in the speed channel of $X_{t}$ are replaced only by training-period regional means and the coverage channel remains available to indicate sensing support.

The primary prediction model was GRU-Mask, which consumed regional speed and coverage channels. It used a hidden size of 96, dropout of 0.1, batch size of 512, learning rate of 10^−3^, and early stopping with a patience of 10 epochs (maximum 120 epochs in the final multi-seed runs). Its recurrent updates were

(5) $$z_{t}=\sigma \left(W_{z}x_{t}+U_{z}h_{t-1}+b_{z}\right)$$

(6) $$r_{t}=\sigma \left(W_{r}x_{t}+U_{r}h_{t-1}+b_{r}\right)$$

(7) $${\tilde{h}}_{t}=\tanh\left(W_{h}x_{t}+U_{h}\left(r_{t}⊙h_{t-1}\right)+b_{h}\right)$$

(8) $$h_{t}=\left(1-z_{t}\right)⊙h_{t-1}+z_{t}⊙{\tilde{h}}_{t},\;\;\;\;\;\;{\hat{y}}_{t+1}=W_{o}h_{t}+b_{o}$$

In Equation (8), $x_{t}$ contains the concatenated regional speed and coverage features, σ(·) is the logistic sigmoid, and ⊙ denotes element-wise multiplication. The masked training objective was

(9) $$L_{MAE}=\frac{\sum_{i=1}^{N}M_{i}\left|y_{i}-{\overset{ˆ}{y}}_{i}\right|}{\sum_{i=1}^{N}M_{i}}$$

where $y_{i}$ and ${\overset{ˆ}{y}}_{i}$ are the observed target speed and prediction, respectively. Final GRU-Mask results were obtained over three random seeds (42, 123, and 2026) on Apple Silicon Metal Performance Shaders (MPS; Apple Inc., Cupertino, CA, USA).

Historical-average, persistence, moving-average, ridge-regression, and histogram gradient boosting (HistGB) were evaluated on the same chronological splits and target masks. HistGB follows the gradient-boosting formulation of Friedman [47]. Ridge used standardized enhanced features with α=1.0. For every target region, the enhanced feature vector comprised the flattened 12-window speed and coverage histories; last, mean, and (for speed) standard-deviation values; the speed change from the first to the last input window; adjacency-normalized neighbor last/mean speed and coverage; and sine/cosine time-of-day and day-cycle features plus a normalized time index. HistGB was fitted separately for each target region using only observed training targets, with squared-error loss, learning rate 0.04, 350 maximum iterations, 31 maximum leaf nodes, minimum leaf size 20, L2 regularization 0.05, a 15% validation fraction for early stopping, and a 25-iteration no-change criterion. If a region had no observed training target, its training-period regional mean was used as the documented fallback. The internal 15% validation fraction used for HistGB early stopping is drawn from the training partition (random split within training); it does not use validation- or test-period observations. Because HistGB consumes the richer enhanced feature set, this comparison is a feature-rich non-neural baseline assessment rather than a strictly architecture-matched comparison.

### 3.5. Deterministic Mask/Coverage-Aware Assessment

**Figure 3:**
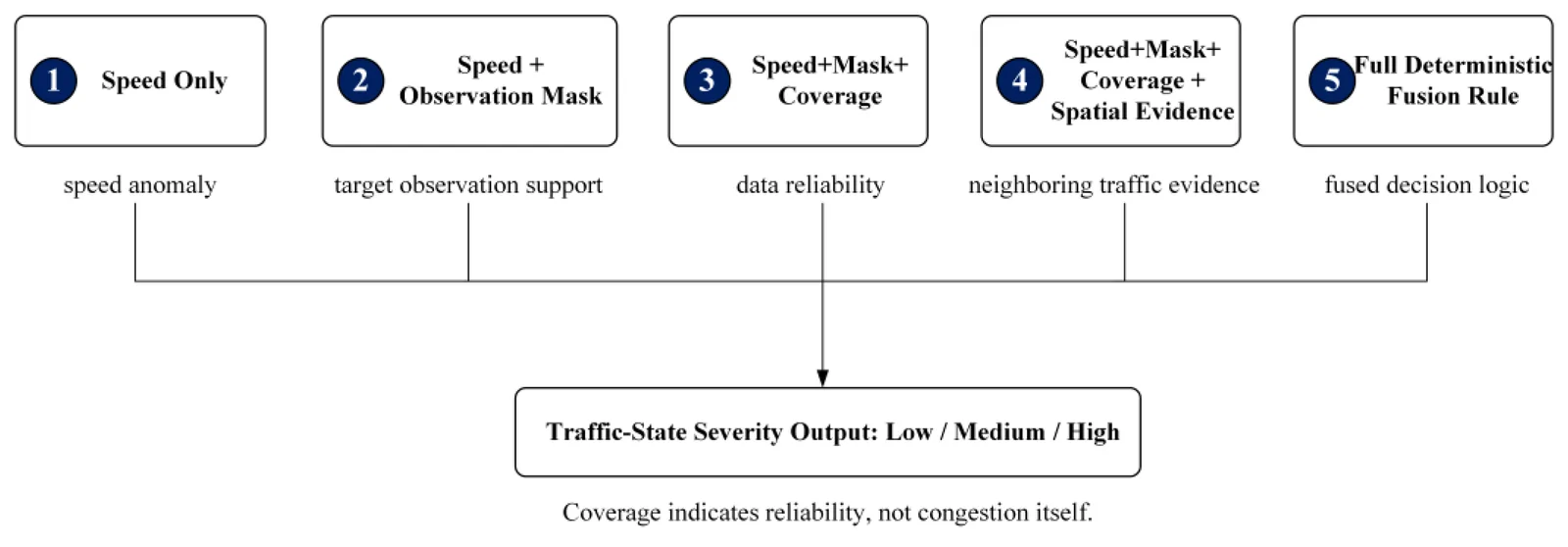
Detailed expansion of Figure 1 module 5. The five deterministic variants progressively add temporal, sensing-quality, and neighboring-region evidence to the speed-only baseline; the fifth is the full deterministic fusion rule. The output is ordinal traffic-state severity (low, medium, or high), not road-safety risk, and the separate LLM stability branch is not included.

Figure 3 visualizes the five deterministic assessment-rule variants implemented as the progressive expansion of Figure 1 module 5: speed only; speed plus temporal mask/history; speed, mask, and coverage; speed, mask, coverage, and spatial evidence; and a full deterministic fusion rule. Each variant assigned one of three ordinal labels (low, medium, or high) without using human labels during rule construction.

Throughout the remainder of the paper, the fifth variant is termed the full deterministic fusion rule (shortened to the full rule where space is limited). It is a fixed threshold-based rule and contains no LLM component.

The initial ordinal score was determined from predicted speed: speeds below 30 km/h, 30–50 km/h, and at least 50 km/h corresponded to high, medium, and low initial severity, respectively. In variants using temporal evidence, a speed drop of at least 35 km/h increased severity when predicted speed was below 55 km/h. A drop of at least 45 km/h after an exceptionally high previous speed (>90 km/h) decreased severity when the predicted speed was at least 35 km/h, thereby controlling inflated drop signals. In coverage-aware variants, a high-severity score was downgraded when current coverage was below 0.001, two or more historical windows had zero coverage, or a sparsity warning was present. Conversely, low-speed evidence was strengthened when mean historical coverage was at least 0.004 and no historical window had zero coverage.

Let $g\left(\hat{v}\right)$ be the initial score from predicted speed $\hat{v}$, with low, medium, and high traffic-state severity encoded as 0, 1, and 2. The assessment structure shared by the ablations is

(10) $$g\left(\overset{ˆ}{v}\right)=\{\begin{array}{cc} 2, & \overset{ˆ}{v}<30,\\ 1, & 30\leq \overset{ˆ}{v}<50,\\ 0, & \overset{ˆ}{v}\geq 50, \end{array}\;\;s={\mathrm{clip}}_{\left[0,2\right]}\left(g\left(\overset{ˆ}{v}\right)+\Delta_{temp}+\Delta_{cov}+\Delta_{spatial}\right).$$

The five variants selectively set ${∆}_{temp}$, ${∆}_{cov}$, and ${∆}_{spatial}$ to zero, so their differences are attributable to the evidence added rather than to a change in the label scale.

Spatial evidence was derived from neighboring regions. A neighbor was considered supportive when it was slow or congested, or had predicted or latest observed speed below 35 km/h, and its historical correlation with the target region was at least 0.03. Two or more supportive neighbors increased severity. The full rule further prevented a high-severity result under sparse data when no supporting neighbor existed. These threshold-based rules are not claimed to be universally optimal; they provide transparent and reproducible evidence-fusion baselines for comparison with the adjudicated reference labels. All rule parameters (30/50/35 km/h speed thresholds, coverage 0.001/0.004, correlation 0.03, and two supporting neighbors) were prespecified expert design choices fixed before label inspection; they are not values calibrated on the adjudicated labels. To make the small coverage thresholds interpretable, for a region of about 1500 analysis segments (the average region size), coverage 0.001 corresponds to roughly one or two observed segments in a 15 min window, and coverage 0.004 to roughly six.

The same prespecified 243-configuration grid was also applied post hoc to the speed-plus-mask and speed-plus-mask-plus-coverage variants as a robustness audit, not as a tuning procedure. Speed plus mask depends only on the low-speed, medium-speed, and large-drop thresholds; the coverage-aware rule additionally depends on the coverage threshold. Consequently, inapplicable coverage or neighbor-count settings create duplicate predictions within the common grid, leaving 15 and 36 distinct prediction vectors, respectively. The predefined configuration remained the reported primary configuration, and no grid result was used for model selection.

More formally, the number of supportive neighbors for target region r is

(11) $$N_{r}^{+}=\sum_{q\in N\left(r\right)}⊮\left[e_{q}=1\wedge {Corr}_{r,q}\geq 0.03\right],\;\;\;\;\;\;\;\Delta_{spatial}=⊮\left[N_{r}^{+}\geq 2\right]$$

where $e_{q}=1$ when neighbor q is labeled slow or congested, or its predicted or latest observed speed is below 35 km/h. The full rule applies an additional high-severity safeguard when the sparse-data indicator is positive and $N_{r}^{+}=0$.

### 3.6. Human Annotation and Adjudication Protocol

Reference labels were created for 130 region–time cases. The set combined an initial 120-case package, balanced across initial-severity levels (40 low, 40 medium, 40 high) and coverage bands (40/40/40), with 10 supplementary cases selected from the under-represented high-severity/high-coverage stratum to improve stratum coverage; the low-severity/low-coverage stratum was structurally unavailable. Case selection was completed before annotation and before any deterministic-rule or LLM evaluation output was produced, and used only pre-label initial-severity and coverage characteristics. Each annotation case included the regional speed and coverage history through t, the target-region and neighboring one-step predictions for t+1, coverage statistics, and neighbor summary. To create an offline human-grounded reference rather than to simulate the prospective system input, annotators were additionally shown the target-observation status and, when the target was observed, its true speed at t+1. The resulting labels are therefore retrospective, human-grounded reference labels created with access to target-time truth; they are not generated under the same information set available to the prospective assessment components. Two annotators independently assigned low, medium, or high operational traffic-state labels, a confidence score from 1 to 4, an uncertainty flag, affected-neighbor regions, and a concise evidence note. They were instructed that sparse coverage alone does not imply high severity; a high label requires consistent speed, temporal, and/or spatial evidence.

**Figure 4:**
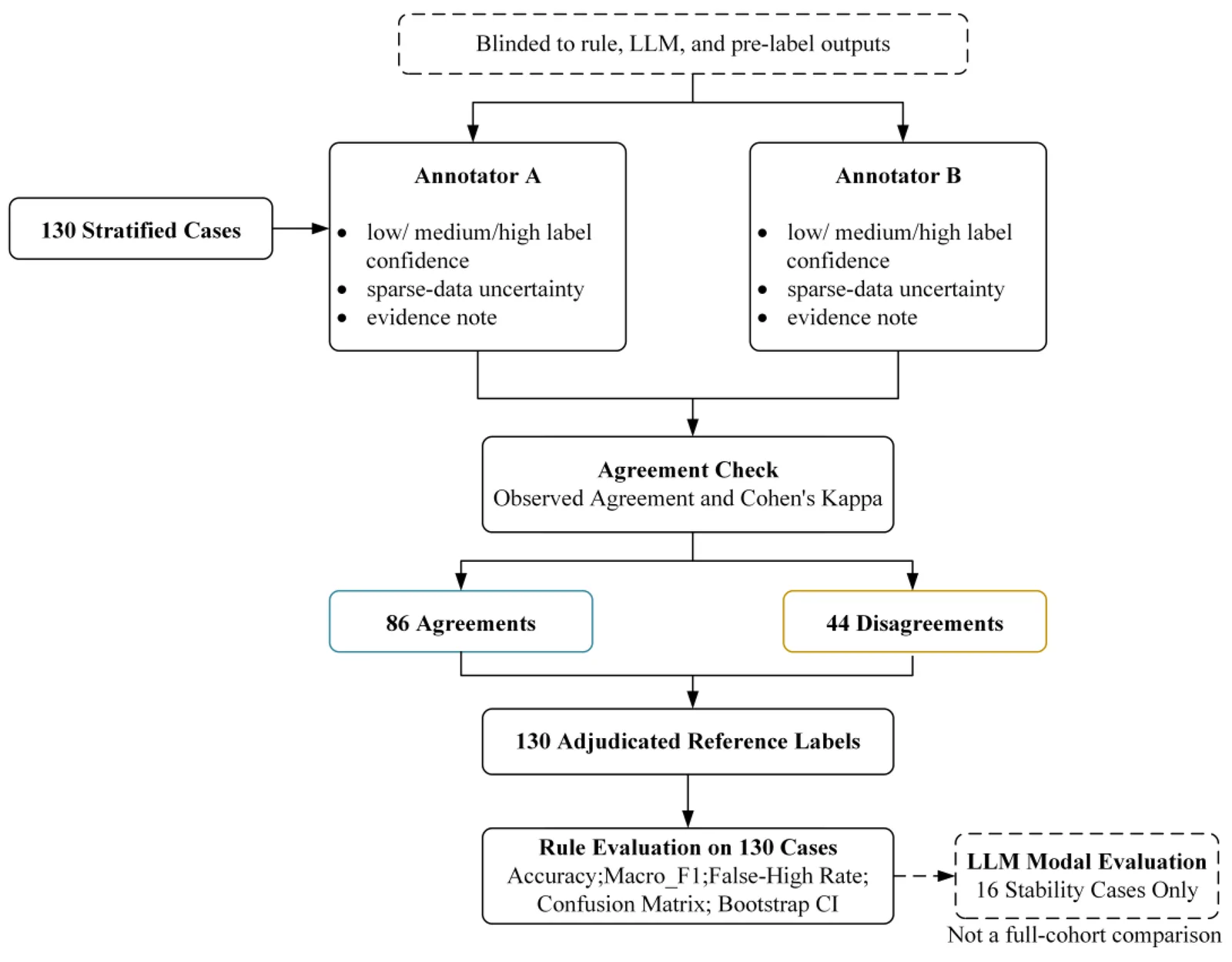
Blinded human annotation, disagreement adjudication, and final evaluation protocol. The rule and LLM outputs were not shown during independent annotation.

As shown in Figure 4, the evaluation workflow began with two independently completed, blinded annotation files before agreement was calculated, moved to disagreement-only adjudication, and ended with offline comparison against the final adjudicated labels. The two labels and their associated case evidence were then reviewed only for disagreements, and a final adjudicated label was recorded for every case. The deterministic rule outputs, LLM outputs, and pre-label behavior analyses were kept out of the annotation workflow. Disagreements were adjudicated by the two annotators using a blinded conflict worksheet that excluded their original labels and confidence values. Conversely, the target true speed and target-observation status used in offline annotation were excluded from deterministic-rule decisions and removed from every LLM input. This separation prevents reference labels from being adjusted toward a system prediction while preventing prospective assessment components from accessing target-time truth.

Of the 130 reference cases, 99 had an observed target-time regional speed and 31 did not. The corresponding adjudicated-label distributions were 61 low, 16 medium, and 22 high for observed targets, versus 29 low, 1 medium, and 1 high for unobserved targets. Target-observation status and target-time speed were available only to the retrospective human-reference workflow and remained excluded from every prospective rule, learned comparator, and LLM input.

### 3.7. LLM Stability Protocol

The LLM analysis was designed as a protocol-stability evaluation—a protocol audit rather than a model-validation experiment—and is not a full-cohort replacement for the deterministic assessments. Sixteen cases were selected from available initial-severity × coverage strata; the low-severity/low-coverage stratum was structurally unavailable under the initial case-selection rule. Each case was evaluated five times at temperature 0 using the DeepSeek V4 Flash model through the DeepSeek API. The recorded run was executed on 13 July 2026 (UTC) through https://api.deepseek.com, with a 1200-token response limit and a 90 s call timeout. The provider/model alias is recorded in the retained run manifest; because the provider did not return an immutable model revision in the retained traces, the results are identified by this alias, endpoint, date, frozen prompts, and saved request/response audit. The run is therefore a date-stamped protocol audit rather than a fully reproducible model experiment. Every repetition used four structured stages: a data-quality agent, a region-state agent, a neighbor-evidence agent, and a coordinator that returned the global traffic-state severity level.

The input excluded target true speed and target-observation outcome. Prompts were fixed across repetitions and every response was checked against a predefined schema. For each case, the modal global-severity label and its repetition ratio were calculated. LLM-derived classification metrics are reported only on these 16 cases and are never presented as directly comparable with the 130-case deterministic-rule evaluation.

For case i with K=5 repetitions and LLM labels $\ell_{i,1},...,l_{i,K}$, the reported label and modal-label repeatability are

(12) $$\ell_{i}^{modal}=\arg\underset{c\in \left\{\mathrm{low},\mathrm{medium},\mathrm{high}\right\}}{\max}\sum_{k=1}^{K}⊮\left[\ell_{i,k}=c\right],\;\;\;\;\;\;\;q_{i}=\frac{1}{K}\underset{c}{\max}\sum_{k=1}^{K}⊮\left[\ell_{i,k}=c\right]$$

### 3.8. Evaluation Metrics and Statistical Analysis

Prediction performance was evaluated only for observed targets using MAE and root mean squared error (RMSE). For assessment, the five fixed deterministic variants, a training-fold majority baseline, balanced multinomial logistic regression, and a balanced RBF-SVM were compared on the same 130 adjudicated labels; learned-model estimates used repeated grouped out-of-fold predictions. We report accuracy, Macro-F1, FSR, false-severe rate among true low-severity labels, under-escalation rate among true high-severity labels, and mean ordinal distance after encoding low, medium, and high as 0, 1, and 2. Accuracy and FSR are defined as

(13) $$Accuracy=\frac{1}{n}\sum_{i=1}^{n}⊮\left[y_{i}={\hat{y}}_{i}\right],\;\;\;\;\;\;\;FSR=\frac{\sum_{i=1}^{n}⊮\left[y_{i}\neq high\wedge {\hat{y}}_{i}=high\right]}{\sum_{i=1}^{n}1\left[y_{i}\neq high\right]}$$

For observed prediction targets, RMSE and MAE are

(14) $$RMSE=\sqrt{\frac{\sum_{i=1}^{N}M_{i}{\left(y_{i}-{\hat{y}}_{i}\right)}^{2}}{\sum_{i=1}^{N}M_{i}}},\;\;\;\;\;\;\;MAE=\frac{\sum_{i=1}^{N}M_{i}\left|y_{i}-{\hat{y}}_{i}\right|}{\sum_{i=1}^{N}M_{i}}$$

For a class set C=low,medium,high, Macro-F1 is calculated from class-specific precision $P_{c}$ and recall $R_{c}$ as

(15) $$Macro\text{-}F1=\frac{1}{\left|C\right|}\sum_{c\in C}\frac{2P_{c}R_{c}}{P_{c}+R_{c}}$$

Inter-annotator reliability was summarized with observed agreement $p_{o}$ and Cohen’s kappa (κ), where $p_{e}$ is the agreement expected from the annotators’ marginal label distributions:

(16) $$\kappa =\frac{p_{o}-p_{e}}{1-p_{e}},\;\;\;\;\;\;\;p_{o}=\frac{1}{n}\sum_{i=1}^{n}1\left[a_{i}=b_{i}\right]$$

To add model-based traffic-state assessment comparators, we constructed a 12-feature prospective design matrix from predicted speed; latest observed speed and speed drop; current, mean, and minimum historical coverage; zero-coverage count; sparsity warning; the numbers of available and supporting neighbors; and the mean and maximum stored neighbor correlations from the frozen evidence package. The response was the adjudicated low/medium/high label. Target true speed, target-observation outcome, annotator labels, confidence and notes, adjudication notes, coverage band, initial rule label, case/region identifiers, and target time were excluded from the predictors. A post hoc correlation-provenance sensitivity audit repeated all deterministic and learned comparisons after replacing the stored correlation values with training-partition estimates; case identities, predictions, histories, neighbor identities and order, and labels remained fixed.

The learned comparators were class-balanced multinomial logistic regression and a class-balanced RBF-SVM, each fitted after training-fold median imputation with missingness indicators and standardization. They were evaluated with 25 repetitions of four-fold StratifiedGroupKFold (seeds 20260920–20260944), grouping by sample_index so regions from the same target time could not cross training and test partitions. No hyperparameter was tuned on the 130-case cohort. Every repeat yielded one out-of-fold prediction for each case, and all preprocessing was fitted within the training fold.

Nonparametric 95% intervals in the original deterministic stratified analyses were estimated from 1000 case-level bootstrap resamples with seed 42. For the expanded same-cohort comparison in Section 4.5, intervals were recomputed using 2000 paired cluster-bootstrap resamples of sample_index groups with seed 20260920; for learned comparators, each bootstrap statistic was averaged across the 25 repeat-specific out-of-fold prediction sets. Metrics were additionally stratified by coverage band, source package, and initial-traffic-state severity level. The LLM modal-label metrics use their actual support of 16 cases and are reported separately.

A post hoc label-uncertainty sensitivity analysis repeated the assessment metrics on three restricted subsets: the 86 direct-agreement cases, the 48 cases for which both annotators assigned confidence at least 3 on the four-point scale, and the 39-case intersection of these criteria. These subsets were defined only to test dependence on contested or lower-confidence reference labels; they were not used to tune rules, train comparators, or select a preferred method.

### 3.9. Implementation, Hardware, and Reproducibility

The experiment pipeline was implemented primarily in Python 3.11.15 (Python Software Foundation, Wilmington, DE, USA), while the same-cohort assessment and correlation-sensitivity analyses were conducted in Python 3.12; full environment details are provided in Appendix A, Table A1. Controlled-missingness generation, region-feature construction, prediction-dataset preparation, deterministic assessment, annotation validation, adjudication import, and metric calculation were executed as separate scripts that write machine-readable manifests together with their outputs. The original assessment-stratification bootstrap used seed 42; the expanded same-cohort paired group bootstrap and repeated grouped comparison used seed 20260920 and seeds 20260920–20260944, respectively; the controlled-missingness generator used seed 20260713; and the final GRU-Mask summary used seeds 42, 123, and 2026. The neural prediction runs used Apple Silicon MPS. The exact operating-system, Python, package, hardware, model-provider, and runtime information is reported in Appendix A and retained in the archival record.

Table 4 records the configuration needed to reproduce the main experiments. Input datasets, derived matrices, annotation artifacts, prompts, and final CSV metrics are retained as separate versioned artifacts so that each manuscript result can be traced to a run manifest.

## 4. Results

### 4.1. Observation Sparsity and Controlled Missingness

The original segment–time matrix contained 674,600 observed cells among 428,932,800 possible cells (0.157%). Controlled missingness therefore evaluated prediction under an already sparse sensing regime rather than under an artificial dense-data setting. Table 5 summarizes the nine missingness conditions. The realized region-level target observation ratio ranged from 12.1% under high temporal-block missingness to 23.9% under low segment-index-block missingness. At high severity, Table 5 contains 6719 evaluable targets (12.1%) for temporal-block missingness, 10,860 (19.6%) for random missingness, and 11,343 (20.5%) for segment-index-block missingness, a 4624-case difference between the smallest and largest supports.

Support contracted differently across mechanisms as severity increased. The low/medium/high observed-target ratios were 23.8%/22.1%/19.6% for random missingness, 21.8%/17.2%/12.1% for temporal blocks, and 23.9%/22.5%/20.5% for segment-index-block. From low to high severity, the evaluable target count fell by 2327 for random missingness, 5336 for temporal blocks, and 1887 for segment-index-block. Because MAE and RMSE are computed only on observed targets, a stable or lower error under a harsher condition can result from a smaller, mechanism-selected evaluable subset and cannot by itself be interpreted as stronger robustness. The segment-index-block series remains a segment-index-based stress test rather than a verified topology-aware outage.

**Figure 5:**
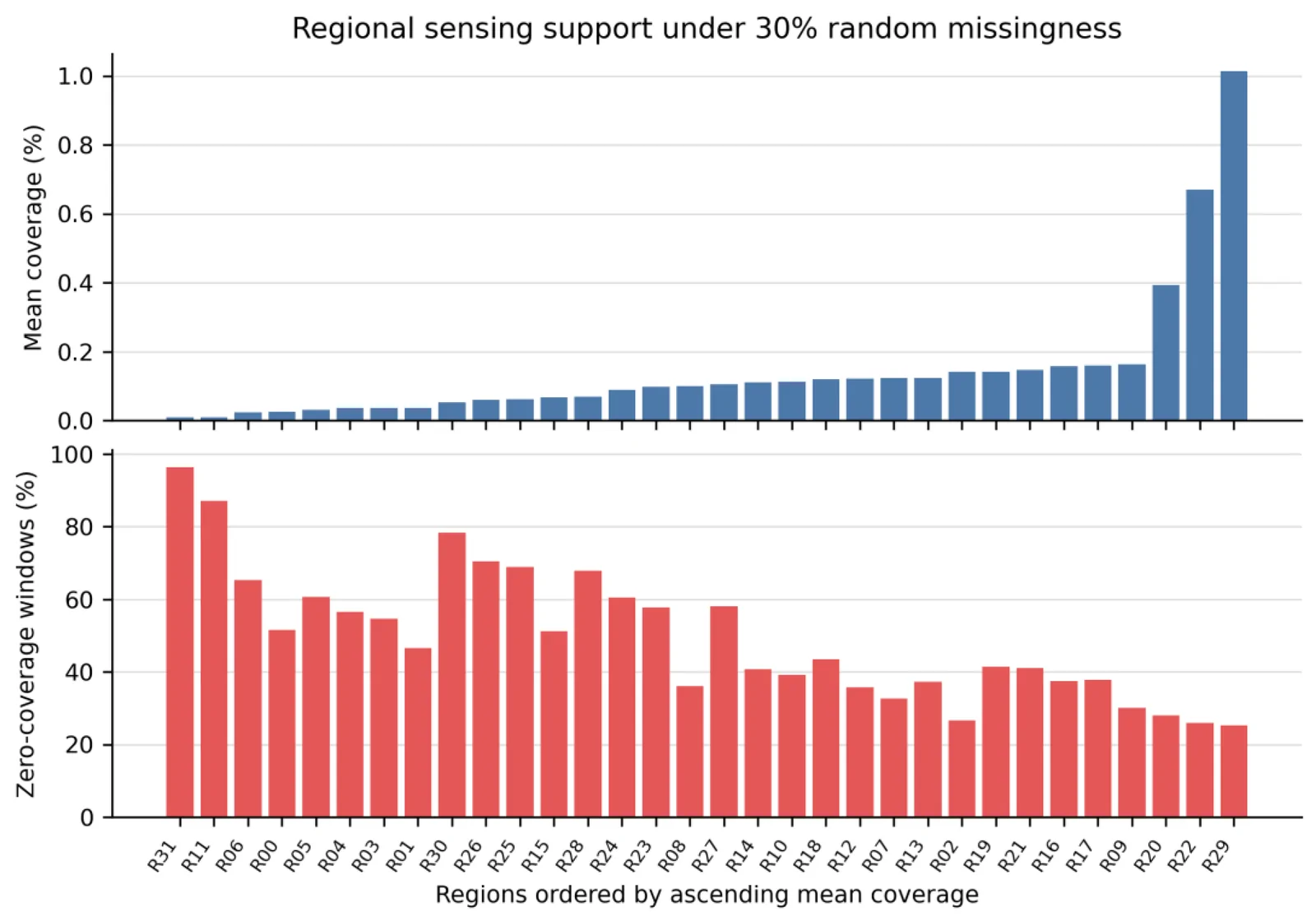
Regional sensing support under the representative 30% random-missingness condition. Regions are ordered by ascending mean coverage; the lower panel reports the share of windows with no observed segment in that region.

**Figure 6:**
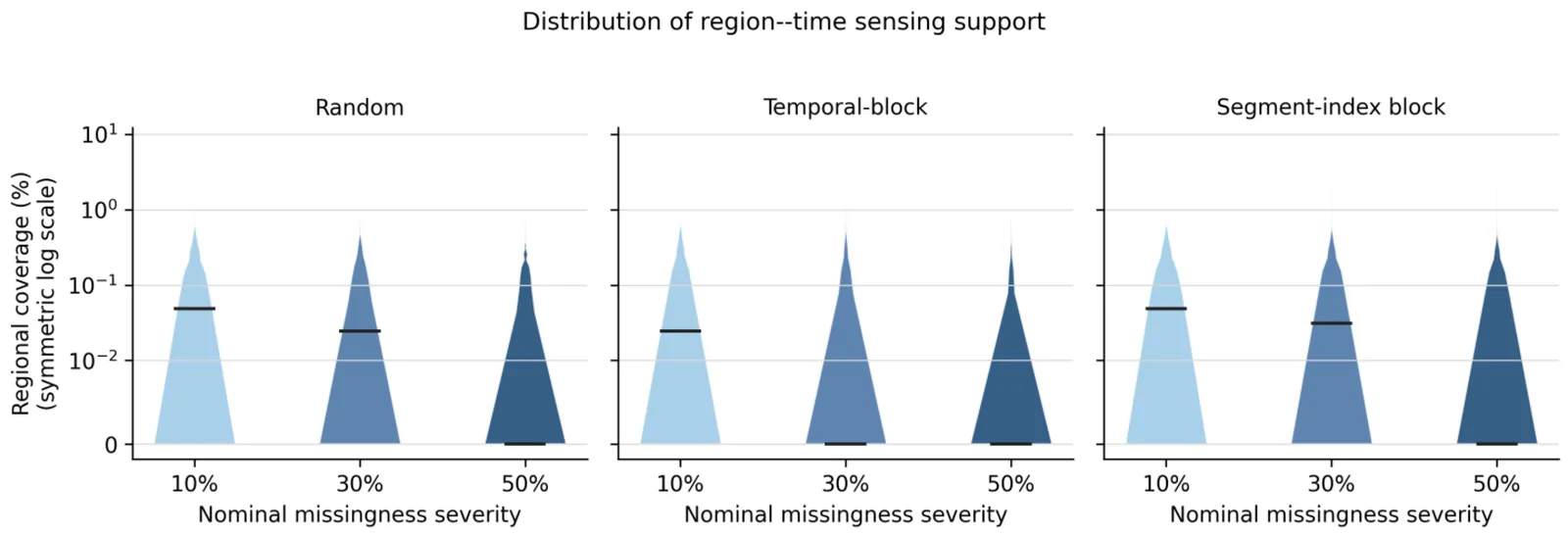
Distribution of regional coverage over all region–time cells for the three controlled-missingness mechanisms and severities. The symmetric logarithmic scale retains both zero coverage and the upper tail of the sparse coverage distribution; the axis is used because the distribution is concentrated near zero.

Figure 5 quantifies this heterogeneity for the representative 30% random-missingness condition. Fifteen of 32 regions had zero coverage in more than half of their windows; overall, 137,318 of 276,480 region–time cells (49.7%) had zero coverage, and region-level mean coverage ranged from 0.0089% to 1.0143%. Figure 6 extends the calculation to all nine conditions: zero-coverage cells ranged from 45.4% to 72.2%. Under high temporal-block missingness, 199,681 of 276,480 cells (72.2%) had zero coverage and all 32 regions were uncovered in more than half of their windows. These distributions show why coverage must remain an explicit reliability input rather than a minor descriptive covariate.

### 4.2. Prediction Under Controlled Missingness

**Figure 7:**
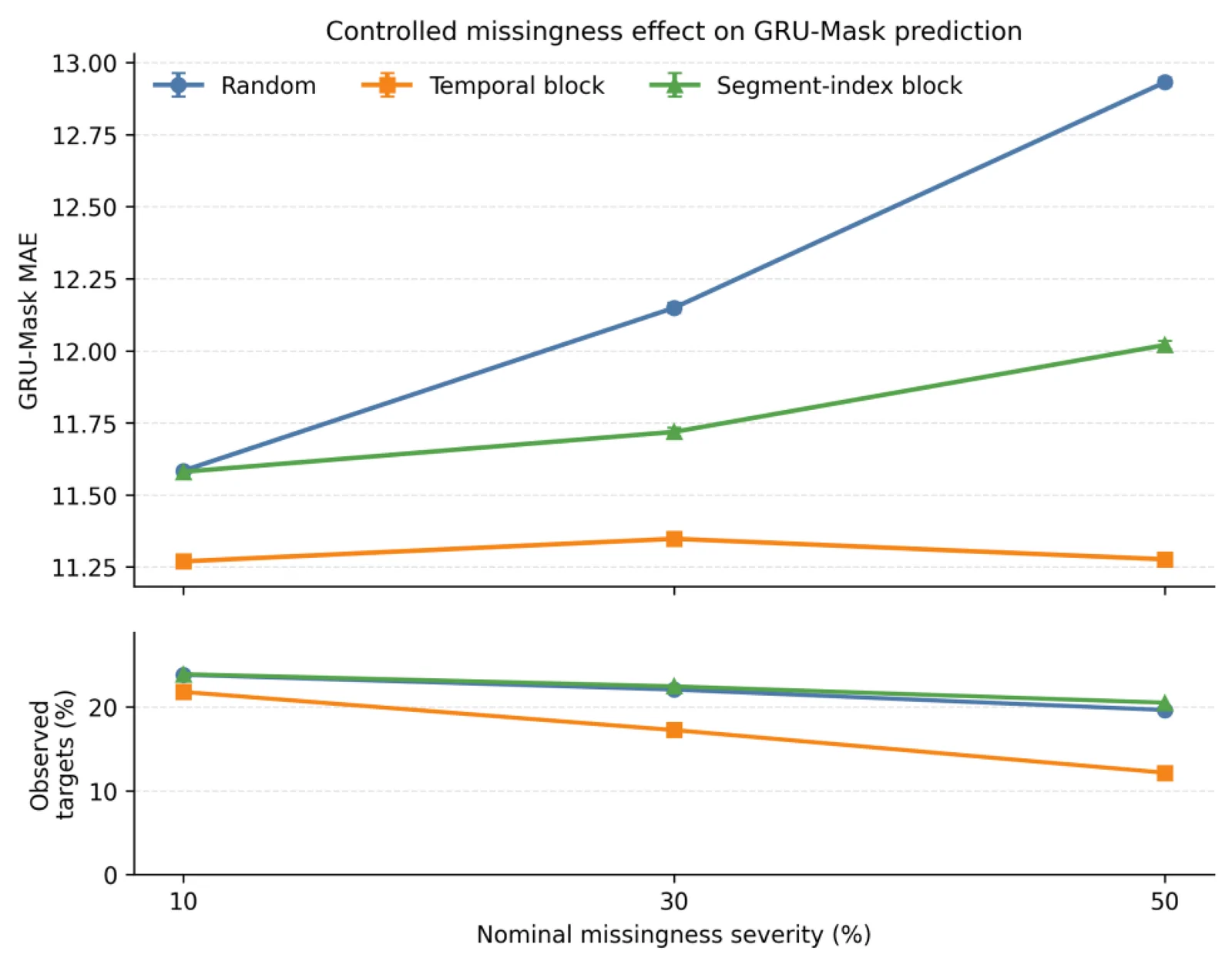
GRU-Mask prediction performance under controlled missingness. The upper panel reports MAE over three seeds; the lower panel reports the observed-target ratio. Error bars indicate one standard deviation across seeds.

Figure 7 shows the GRU-Mask MAE together with the observed-target ratio. GRU-Mask achieved a lower MAE than the historical-average baseline in all nine conditions. Its MAE increased under random missingness from 11.584 km/h at low severity to 12.932 km/h at high severity. Under temporal-block missingness, the MAE remained between 11.269 and 11.347 km/h, but the observed-target ratio declined from 21.8% to 12.1%. Thus, a stable error alone should not be interpreted as unchanged sensing robustness when the evaluable target set has contracted.

### 4.3. Prediction Baseline Comparison

**Figure 8:**
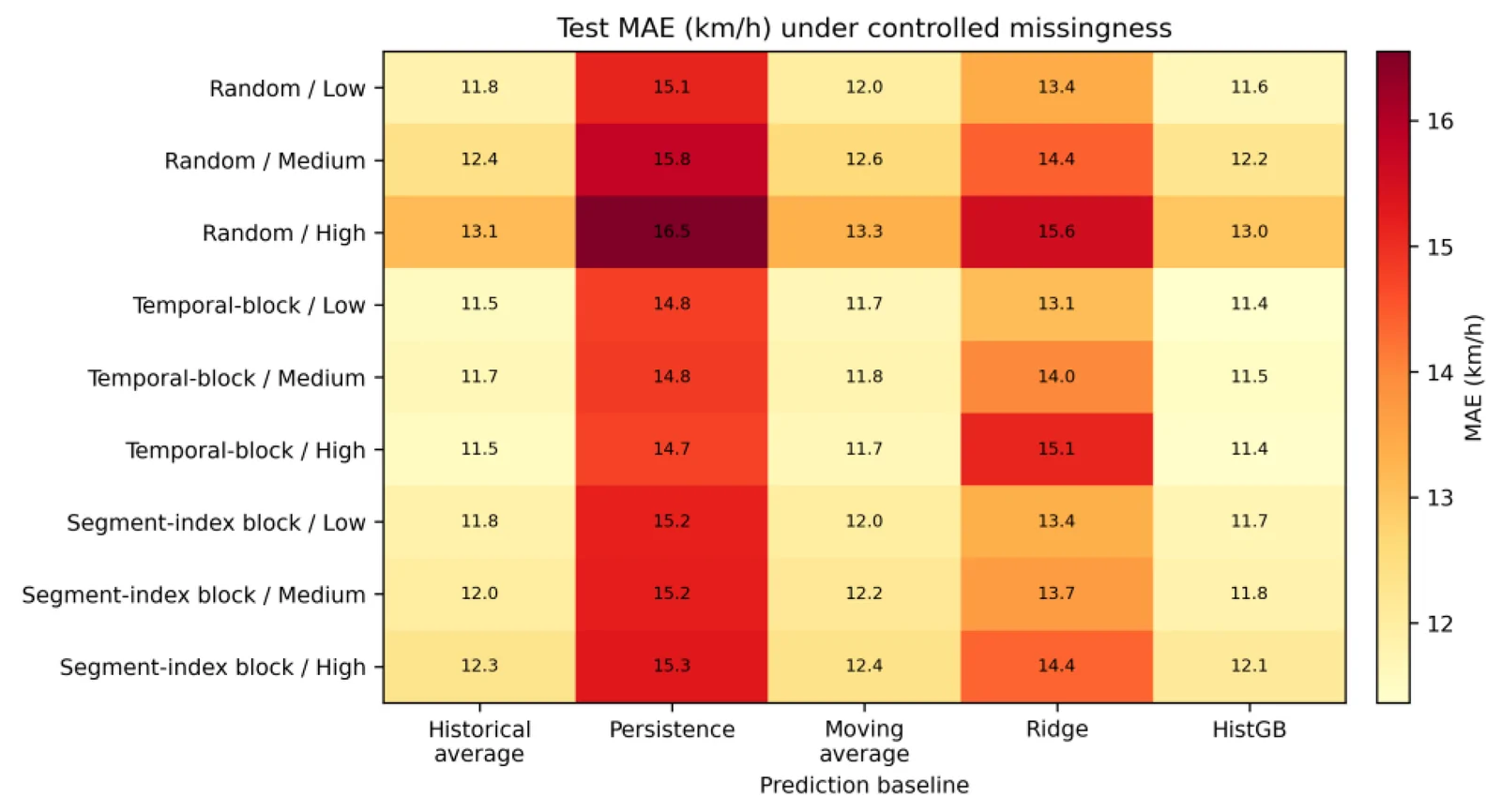
Test mean absolute error (MAE) of five non-neural prediction baselines under the nine controlled-missingness conditions. All values use the same test target masks as the GRU-Mask experiment. Lower values are better.

To determine whether the masked recurrent model was being compared only with a weak reference, four additional baselines were evaluated on exactly the same nine masked datasets and test masks: historical average, persistence, 12-window moving average, ridge regression with mask/coverage features, and histogram gradient boosting (HistGB) with the same enhanced features. Figure 8 reports the complete test-MAE matrix rather than a single average, because the missingness mechanism changes the available support and the relative behavior of the baselines.

HistGB had the lowest mean test MAE across the nine conditions (11.860 km/h; range 11.368–12.985 km/h), followed by historical average (12.024 km/h) and moving average (12.170 km/h). The persistence and ridge baselines were consistently weaker (mean MAE 15.274 and 14.116 km/h, respectively). GRU-Mask remained lower than the historical-average benchmark in every condition (Table 5), while HistGB provides a strong non-neural reference under the present sparse-support setting. These baseline results are descriptive comparisons under the fixed split, not a claim of universally superior prediction performance across traffic networks.

### 4.4. Annotation Reliability and Final Reference Labels

**Figure 9:**
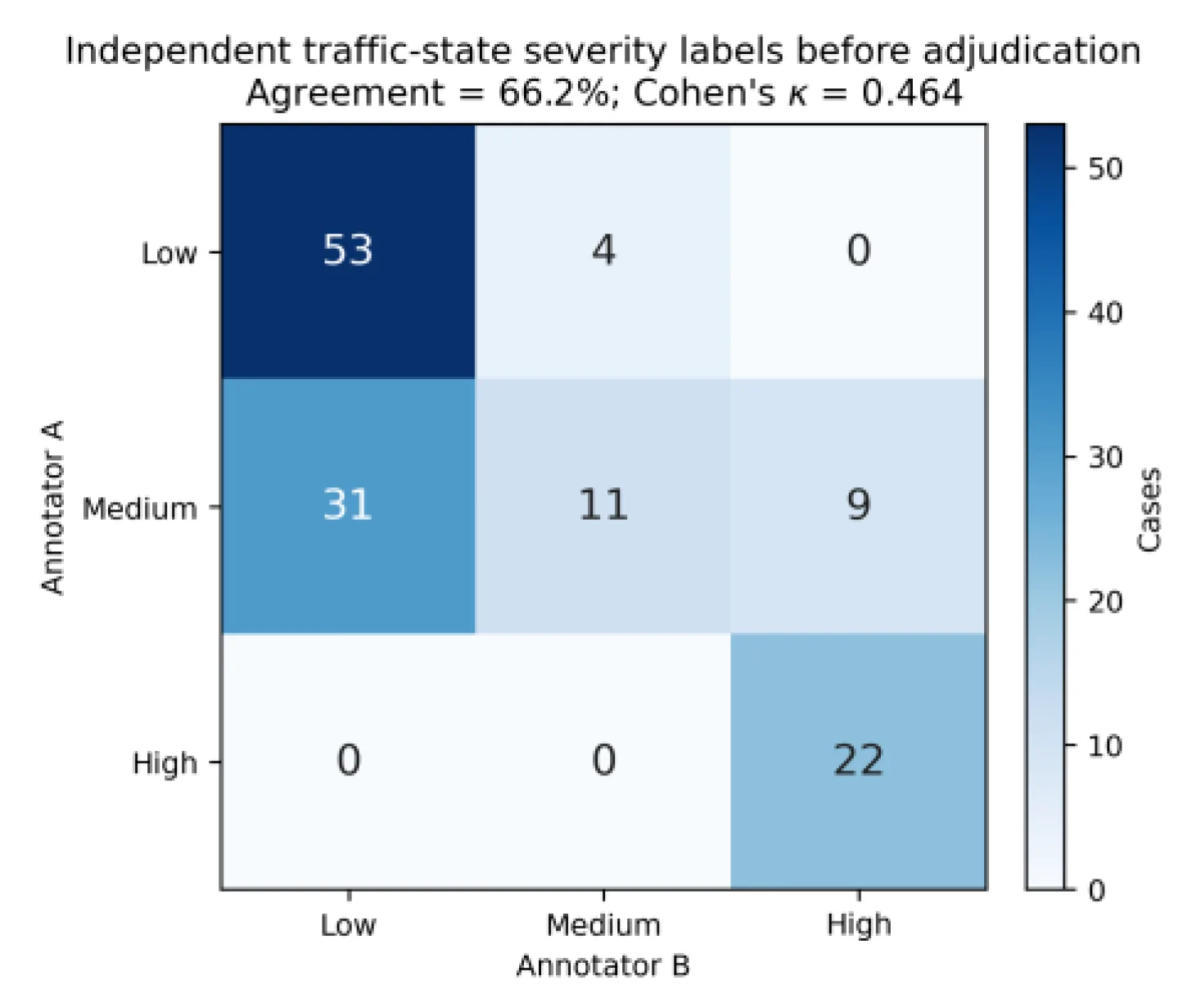
Cross-tabulation of the two independent traffic-state severity labels before adjudication. The concentration of disagreement in the medium-severity labels motivates retaining adjudication and Macro-F1 rather than reporting accuracy alone.

As summarized in Table 6, all 130 cases received two independent labels and a final adjudicated label; the annotators agreed directly on 86 cases (66.2%; Cohen’s κ=0.464), and 44 disagreements were resolved through the predefined adjudication procedure. Figure 9 localizes this uncertainty: all 44 disagreements involved at least one medium-severity label (31 annotator-A medium versus annotator-B low, nine medium versus high, and four low versus medium), whereas no case was a direct low-versus-high disagreement. The uncertainty is therefore concentrated at the ordinal class boundaries, supporting adjudication and Macro-F1 reporting; the final labels remain a human-grounded reference rather than objective ground truth. The final reference set comprised 90 low-, 17 medium-, and 23 high-severity cases. The class imbalance and the small medium-severity class are retained in the reported Macro-F1 values rather than hidden by accuracy alone.

Using symmetric category-specific positive agreement, defined for each class as twice the diagonal count divided by the sum of the two annotator marginals, agreement was 75.2% for low, 33.3% for medium, and 83.0% for high severity. The markedly lower medium-class value quantitatively confirms that uncertainty was concentrated at the two ordinal boundaries rather than between low and high severity. Annotator A used low, medium, and high labels in 57, 51, and 22 cases, whereas annotator B used them in 84, 15, and 31 cases. This pronounced marginal asymmetry—especially annotator B’s much less frequent use of medium—contributes to the low medium-category agreement; the available data cannot distinguish a stable rater-threshold difference from case-specific interpretation uncertainty.

Performance was also stratified by target-time observability. On the 99 target-observed cases, speed plus mask achieved 69.7% accuracy and 65.9% Macro-F1, coverage-aware assessment achieved 60.6% and 60.2%, and the RBF-SVM averaged 74.9% and 67.3%. On the 31 target-unobserved cases, the corresponding values were 67.7%/32.8%, 51.6%/26.6%, and 75.5%/38.6%. Because 29 of the 31 unobserved cases were low severity, the latter Macro-F1 values and apparent accuracy are descriptive rather than a balanced test of target-unobserved performance.

The label-uncertainty audit did not preserve a single ranking across all restricted subsets. On the 86 direct-agreement cases, the RBF-SVM had the highest accuracy (82.4%), whereas speed plus mask had the highest Macro-F1 (72.9%). Among the 48 cases with confidence of at least 3 from both annotators, the RBF-SVM again had the highest accuracy (79.3%) but the coverage-aware rule had the highest Macro-F1 (74.0%). On the strict 39-case intersection, the coverage-aware rule had both the highest accuracy (82.1%) and Macro-F1 (79.3%). These post hoc results show that comparative rankings are sensitive to which human-reference cases are retained and reinforce the need for a larger independently labeled cohort.

### 4.5. Human-Grounded Assessment Performance

Section 4.1, Section 4.2 and Section 4.3 established that prediction error varies with missingness mechanism and evaluation support; assessment reliability, however, depends on how sensing support is fused with predicted speed rather than on MAE alone. Section 4.4, Section 4.5, Section 4.6, Section 4.7, Section 4.8 and Section 4.9 therefore evaluate the assessment rules independently against the adjudicated labels. Table 7 compares the five fixed deterministic variants, a training-fold majority baseline, balanced multinomial logistic regression, and a balanced RBF-SVM on the same 130 adjudicated cases; learned-model estimates use only repeated grouped out-of-fold predictions. Among the fixed deterministic variants, the speed-plus-mask rule achieved the highest accuracy (69.2%) and Macro-F1 (63.7%). A majority-class baseline that always predicts the modal class (low severity) attains the same accuracy by construction (90/130 = 69.2%) but only 27.3% Macro-F1 and 33.3% balanced accuracy; the speed-plus-mask advantage therefore lies in cross-class discrimination rather than in overall accuracy alone. Adding coverage reduced the FSR from 5.6% (6 of 107 non-high reference cases) to 2.8% (3 of 107) and eliminated false-severe classifications among reference low-severity cases, but reduced accuracy to 58.5%. The under-escalation rate remained 4.3% (one of 23 adjudicated high-severity cases) for all deterministic variants; thus, the observed false-severe-rate reduction did not eliminate high-severity misses. This is an over-escalation-control–classification trade-off rather than a universal improvement in every metric. Paired group-bootstrap 95% confidence intervals (Table 7) overlap across the fixed variants (e.g., speed-plus-mask accuracy 69.2% [60.9, 77.3] versus speed-only 68.5% [59.8, 76.4]; FSR 5.6% [1.9, 10.2] versus 2.8% [0.0, 6.4]), so cohort-level differences should be read as directional rather than definitive. Under the training-partition-correlation sensitivity audit, 641 stored neighbor-correlation values changed; the supporting-neighbor count changed in 2 of 130 cases, but none of the 650 deterministic case-variant labels changed. Logistic-regression accuracy/Macro-F1 changed from 74.2%/62.8% to 73.7%/62.5%, whereas RBF-SVM changed from 75.0%/64.6% to 75.2%/65.0%. These small shifts support the stability of the reported ranking to the stored correlation values, but they do not remove the transductive neighbor-selection limitation because neighbor identities and order were held fixed.

The balanced RBF-SVM produced the highest mean grouped out-of-fold accuracy (75.0%, 95% CI 67.6–81.6%) and Macro-F1 (64.6%, 55.3–72.7%), while speed plus mask retained the highest balanced accuracy among the reported methods (69.7%, 58.5–79.9%) and a lower FSR (5.6%, 1.9–10.2%) than the RBF-SVM (9.7%, 4.7–15.5%). Logistic regression yielded 74.2% accuracy and 62.8% Macro-F1. For the four prespecified headline differences—accuracy, Macro-F1, balanced accuracy, and FSR—the paired RBF-SVM-minus-speed-plus-mask 95% intervals all included zero. In the secondary class-specific audit, the RBF-SVM had a higher true-low-to-high rate by 5.4 percentage points (95% CI 0.3–10.5), an RBF-SVM disadvantage, and higher low-class recall by 11.7 points (95% CI 5.0–19.1), an RBF-SVM advantage; its under-escalation interval touched zero. The cohort therefore shows direction-dependent differences rather than uniform superiority, and neither the headline averages nor the class-specific averages alone should determine method selection.

**Figure A1:**
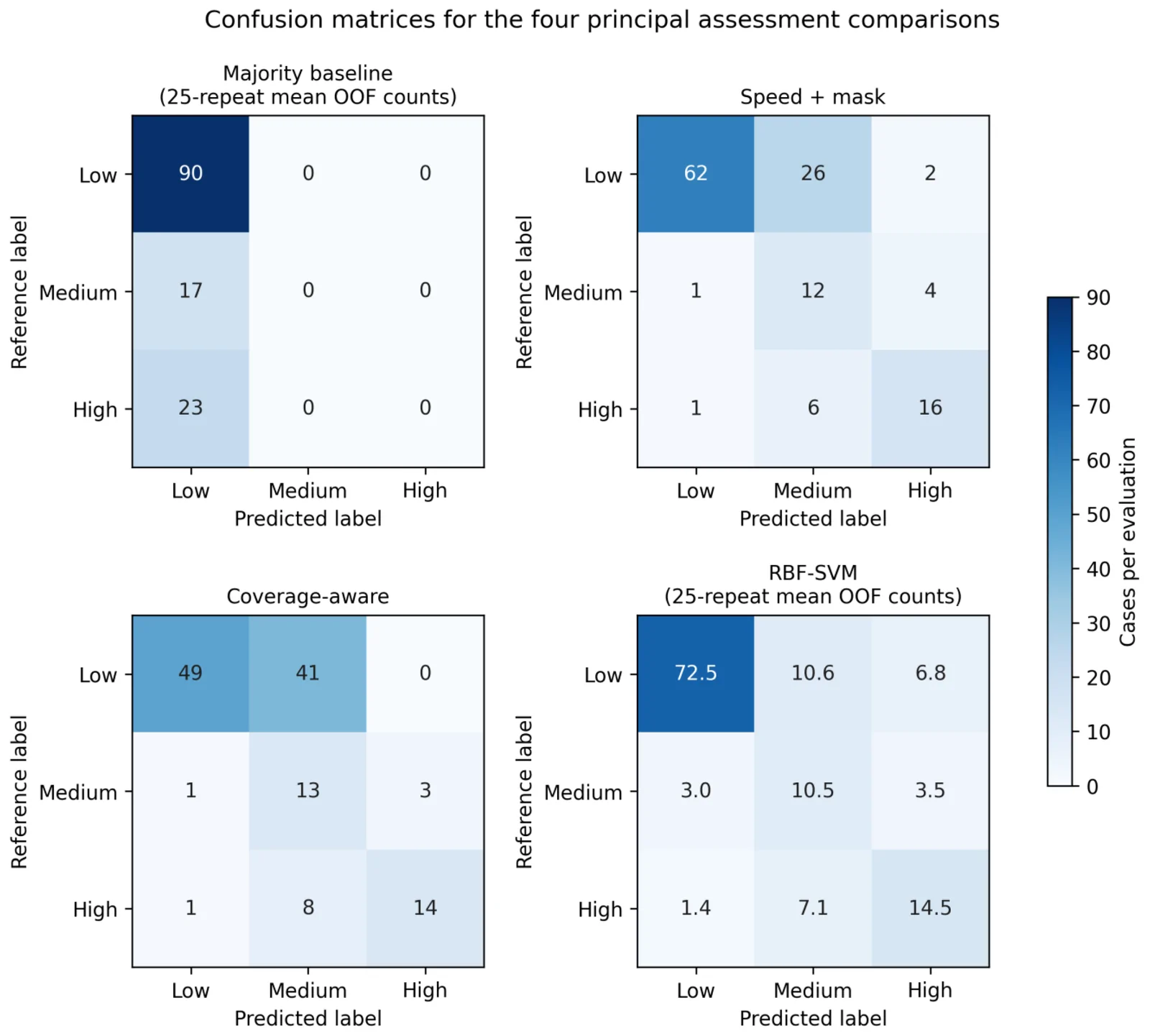
Confusion matrices for the four principal assessment comparisons. Majority, speed plus mask, and coverage-aware cells are exact counts on the 130-case cohort. RBF-SVM cells are mean counts per grouped out-of-fold repetition across 25 repetitions.

Class-specific precision, recall, and F1-score for the four principal comparisons are reported in Table A5, and Figure A1 presents their confusion matrices. Speed plus mask yielded low-, medium-, and high-class F1-scores of 80.5%, 39.3%, and 71.1%; the coverage-aware rule yielded 69.5%, 32.9%, and 70.0%; and the RBF-SVM averaged 86.9%, 46.4%, and 60.6% across the 25 grouped out-of-fold repetitions. The RBF-SVM values in Table A5 are repeat-wise means, and its Figure A1 matrix reports the mean cell count per repetition rather than a single consensus-label matrix.

The full deterministic fusion rule and the spatial variant both had lower accuracy (54.6%) and a substantially higher FSR (28.0%). This result indicates that the present, uncalibrated spatial rule thresholds over-escalated traffic-state alerts in the available cohort: the correlation threshold (0.03) treats almost any weakly positive correlation as supportive, and neighbor support is counted without temporal alignment or topology-aware connectivity. The finding is reported as a limitation of this uncalibrated deterministic baseline, not as evidence that spatial context is intrinsically harmful or that spatial information is not useful after recalibration.

**Figure 10:**
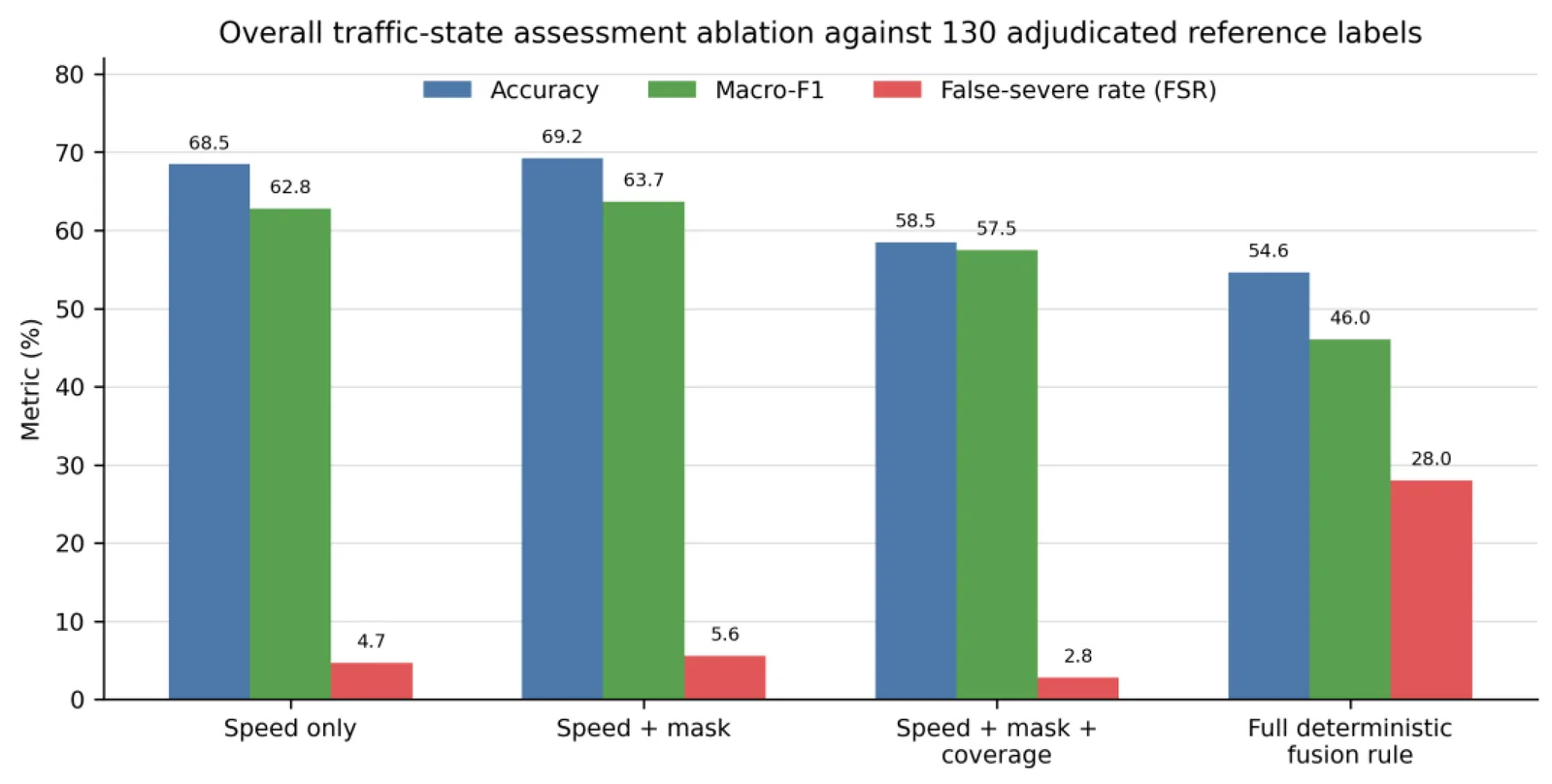
Overall deterministic traffic-state assessment ablation against the 130 adjudicated labels. Among the evaluated variants, adding the observation mask gives the strongest overall accuracy and Macro-F1; adding coverage reduces false-severe traffic-state alerts but entails an overall classification-performance trade-off.

Figure 10 makes the deterministic ablation trade-off explicit. Adding the observation mask to speed increased accuracy from 68.5% to 69.2% and Macro-F1 from 62.8% to 63.7%, although FSR changed from 4.7% to 5.6%. Adding coverage then reduced FSR from 5.6% to 2.8% and eliminated low-to-high errors (2.2% to 0.0%), but accuracy and Macro-F1 fell to 58.5% and 57.5%, respectively. Thus, the mask provides the strongest overall deterministic discrimination in this cohort, whereas coverage acts as a conservative over-escalation control with a measurable discrimination cost.

### 4.6. Coverage-Stratified Results

**Figure 11:**
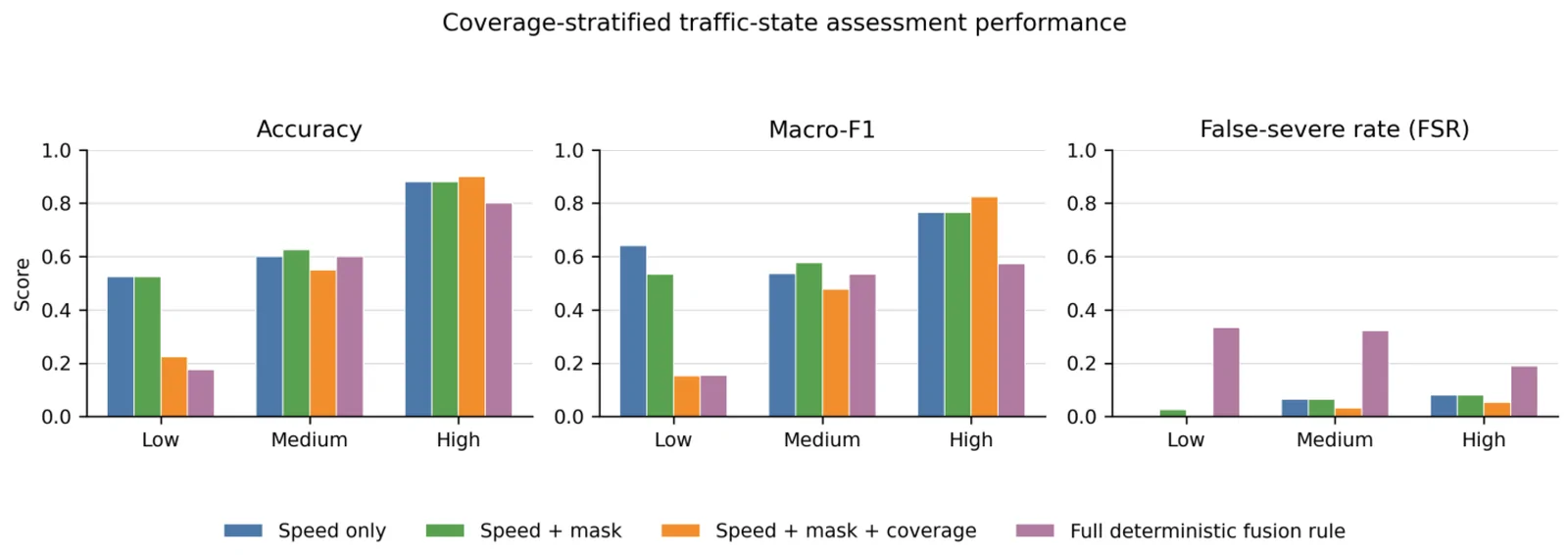
Coverage-stratified performance of deterministic assessment-rule variants. Results use the 130 adjudicated cases: low coverage, n=40; medium coverage, n=40; high coverage, n=50.

Figure 11 stratifies rule performance by coverage band. The high-coverage stratum (n=50) yielded the strongest results across variants; the coverage-aware rule reached 90.0% accuracy, 82.4% Macro-F1, and a 5.4% FSR in this stratum. In the low-coverage stratum (n=40), the same rule reduced FSR to 0.0%, but its accuracy and Macro-F1 were 22.5% and 15.2%, respectively. The suppression did not degenerate to a constant low prediction: within this stratum the rule’s predictions were 34 medium and six low (no high), i.e., high-severity scores were downgraded toward medium; this explains the zero FSR together with the reduced discrimination and should be interpreted as an operational conservative fallback rather than a fine-grained reliability adjustment. This pattern is consistent with the intended conservative behavior under weak sensing support, but it also shows that uncertainty-aware downgrading can reduce discrimination.

The full deterministic fusion rule produced the highest FSR in both low-coverage (33.3%) and medium-coverage (32.3%) strata. Therefore, the coverage analysis does not support treating the current full deterministic fusion rule as the primary assessment configuration. The speed-plus-mask result is the strongest overall deterministic baseline, while the speed-plus-mask-plus-coverage result provides the clearest over-escalation-control comparison.

### 4.7. Threshold Robustness and Coverage-Selective Assessment

**Figure 12:**
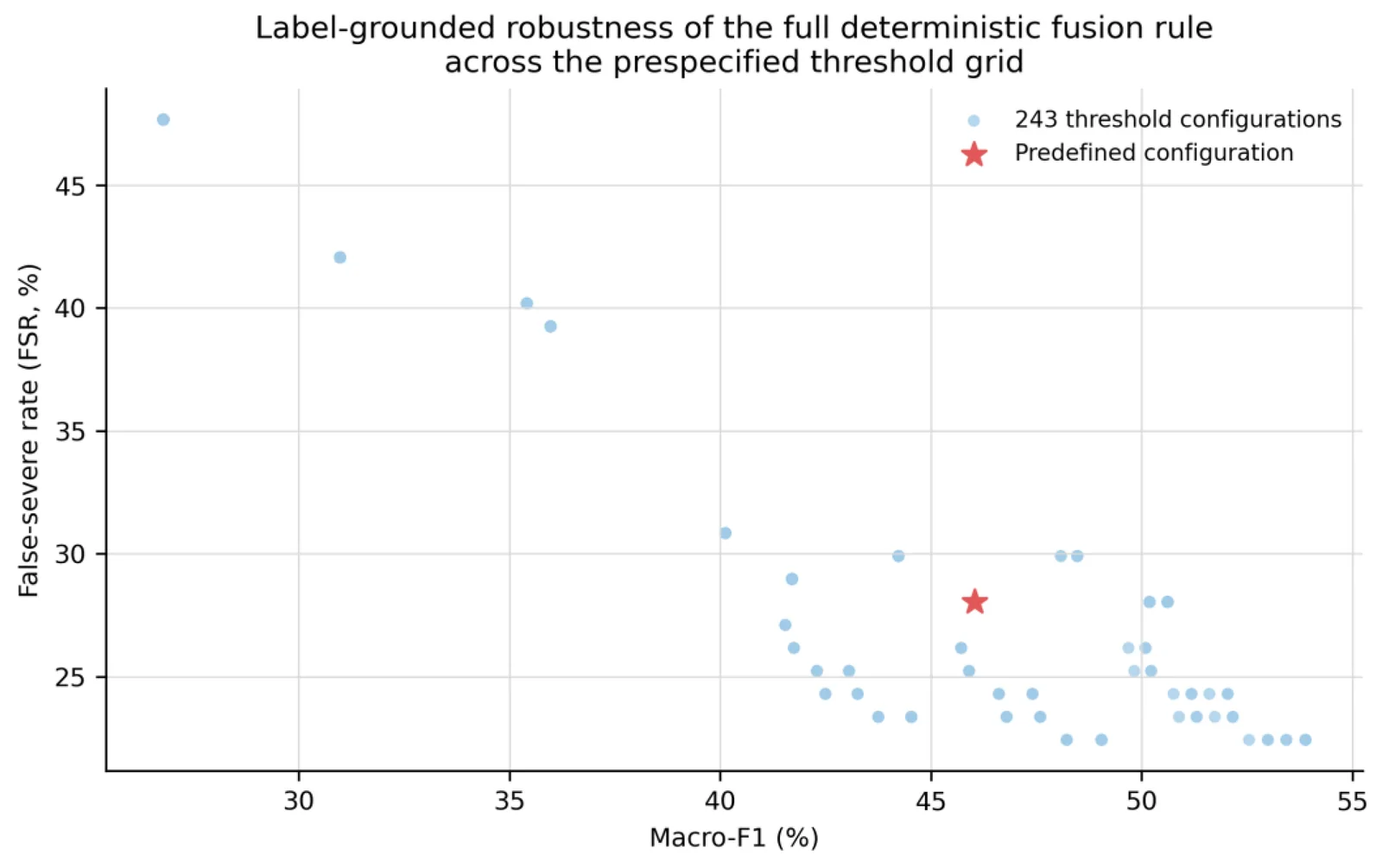
Label-grounded robustness of the full deterministic fusion rule across the prespecified 243-configuration threshold grid. The starred point is the predefined configuration (coverage 0.001; 30/50 km/h thresholds; 35 km/h drop; two supporting neighbors); each point is one of $3^{5}$ = 243 combinations of five rule parameters; the grid is not retuned after observing the labels.

The primary deterministic rule retained its predefined parameterization ($C_{min}$ = 0.001, $v_{slow}$ = 30 km/h, $v_{medium}$ = 50 km/h, ${∆v}_{large}$ = 35 km/h, and two supporting neighbors). To characterize, rather than optimize, rule sensitivity, all 243 configurations ($3^{5}$) in the prespecified grid were evaluated after label adjudication. The grid varied the coverage threshold (0.0005, 0.001, 0.002), the low-speed threshold (25, 30, 35 km/h), the medium-speed threshold (45, 50, 55 km/h), the large-drop threshold (30, 35, 45 km/h), and the supporting-neighbor count (1, 2, 3); these values were fixed before label inspection. The same grid covers the full deterministic fusion rule; Figure 12 shows substantial variation: Macro-F1 ranged from 26.8% to 53.9%, and FSR ranged from 22.4% to 47.7%. The predefined configuration produced 46.0% Macro-F1 and 28.0% FSR. This analysis is therefore evidence that threshold choice materially affects the full deterministic fusion rule; it does not justify retrospectively selecting the best-performing configuration for the reported primary comparison.

Under the same 243 listed configurations, speed plus mask produced 15 distinct prediction vectors, with accuracy ranging from 58.5% to 71.5%, Macro-F1 from 49.6% to 64.5%, and FSR from 0.0% to 25.2%. The coverage-aware rule produced 36 distinct vectors, with accuracy from 49.2% to 66.9%, Macro-F1 from 49.2% to 62.4%, and FSR from 0.9% to 10.3%. At matched listed configurations, speed plus mask had higher accuracy in all 243 comparisons and higher Macro-F1 in 180, whereas the coverage-aware rule had lower FSR in 135 comparisons and never had a higher true-low-to-high rate. These results confirm a persistent discrimination-versus-false-severe-control trade-off; they do not justify replacing the predefined settings with a post hoc optimum.

**Figure 13:**
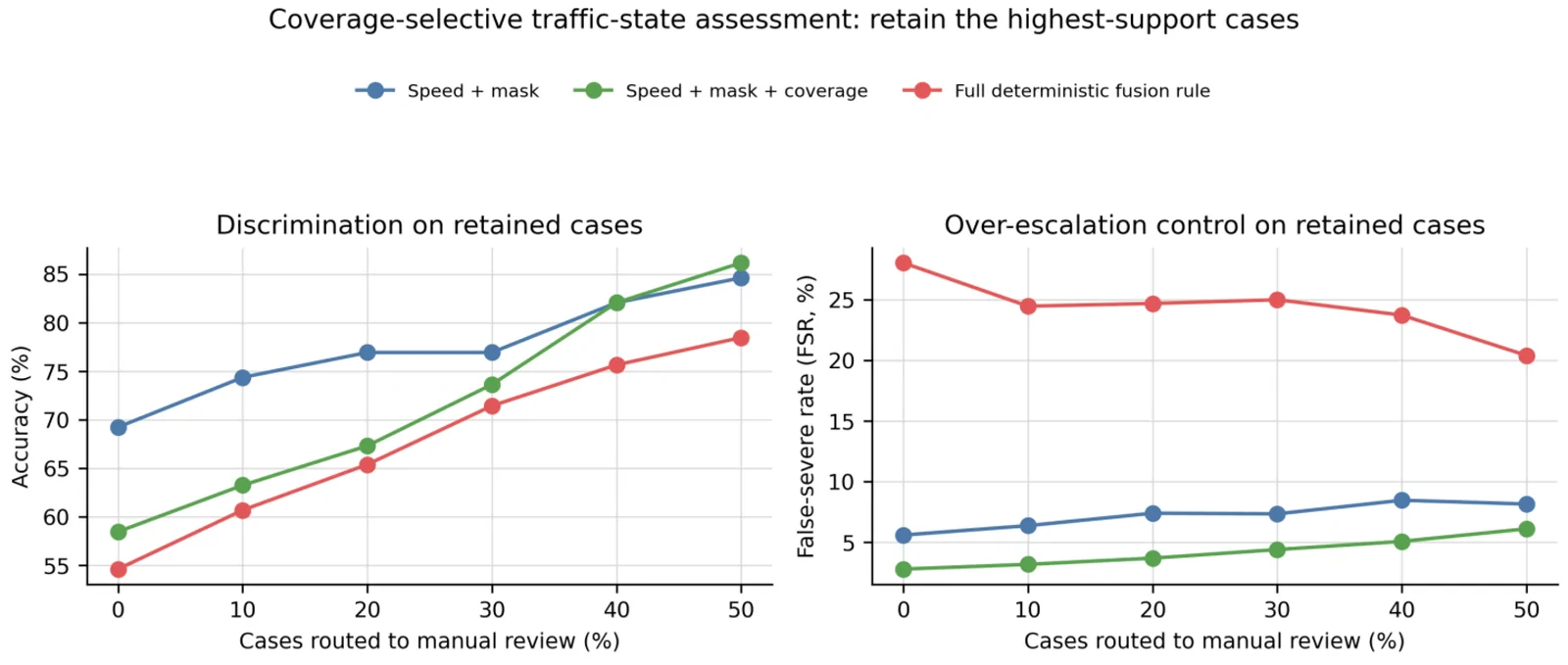
Coverage-selective assessment on the adjudicated cohort. At each point, the lowest-support cases are routed to manual review and the remaining cases are evaluated. The curves demonstrate an operational trade-off and should not be interpreted as a fully evaluated human–machine deployment.

We also evaluated a deployment-oriented selective policy that retains the cases with greatest current observation coverage and routes the rest to manual review. For the speed-plus-mask rule, routing the lowest-coverage 20% of cases to review increased retained-case accuracy from 69.2% (n=130) to 76.9% (n=104); when the lowest-coverage 50% were routed to review, accuracy was 84.6% (n=65 retained). The coverage-aware rule remained more conservative, with FSR 2.8% on the full cohort and 3.7% when the lowest-coverage 20% of cases were routed to review (n=104 retained). Figure 13 presents the full trade-off. These numbers describe retained-set conditional accuracy—selective performance conditional on coverage ranking—not an end-to-end estimate of a deployed human–machine workflow.

### 4.8. Representative Evidence Trace Under Sparse Support

**Figure 14:**
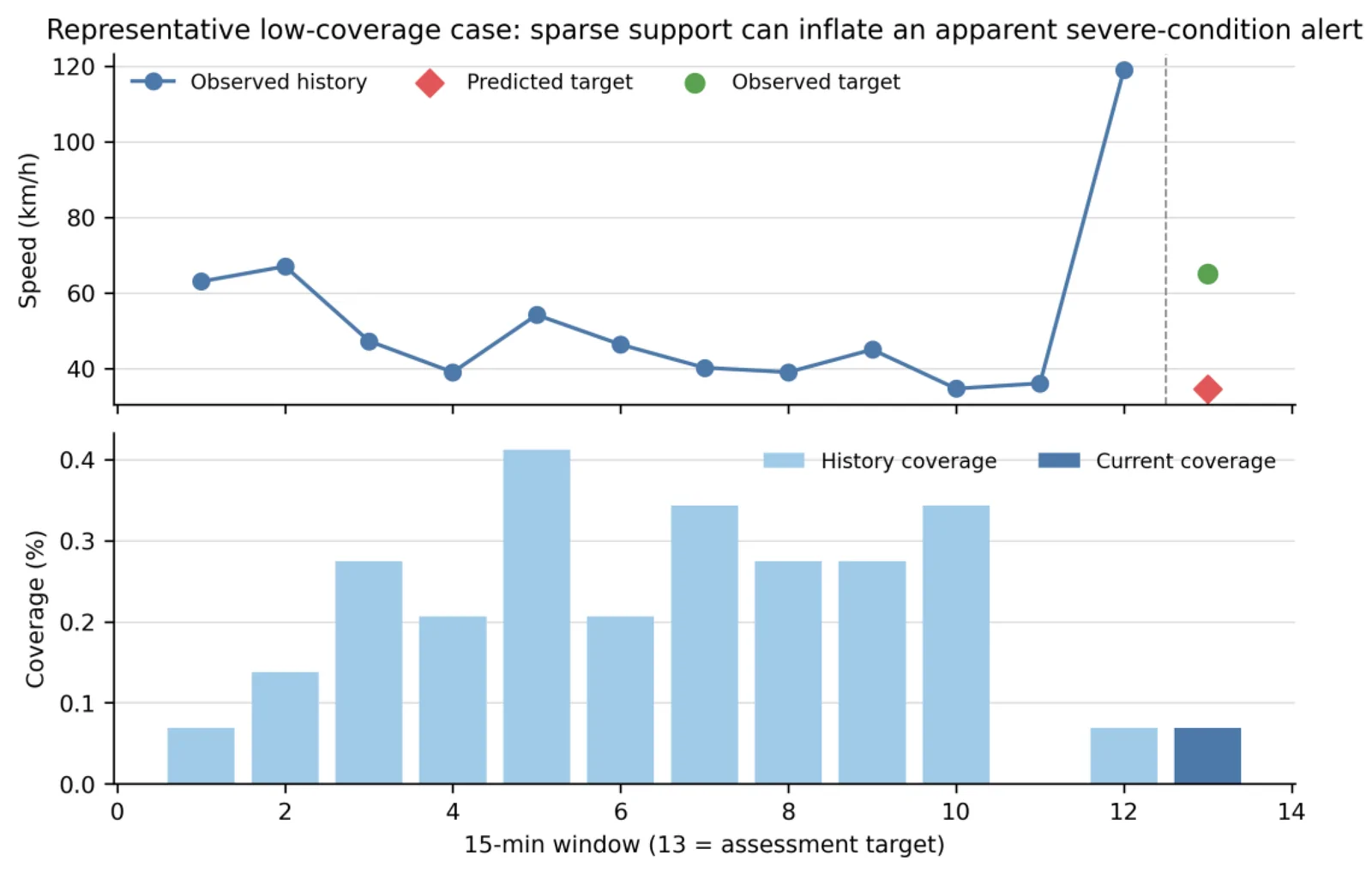
Representative low-coverage case (region 15, 28 March 2026, 18:00). A high last-observed speed and low predicted speed imply a large apparent drop, while the sparse coverage history and the observed target speed support the adjudicated low-severity reference label.

Figure 14 shows a representative low-coverage case that was independently labeled low severity after adjudication. Its last observed speed was 119.0 km/h and the predicted target speed was 34.6 km/h, which produces a large apparent drop. However, the target observation was 65.0 km/h and the coverage history contained a zero-coverage window; current coverage was only 0.069%. The case demonstrates why a speed-drop alert must be interpreted jointly with observation support. It does not establish a universal rule from a single example, but makes the failure mode quantified by the cohort-level FSR interpretable.

### 4.9. LLM Stability and Limited-Sample Evaluation

Table 8 summarizes the LLM stability protocol: 16 cases generated 80 traffic-state assessments through five repetitions and four structured agent calls per repetition. All 320 calls were completed successfully and all outputs satisfied the required schema. The mean call latency was 3.19 s and 15 of 16 cases had perfect modal-label repeatability; the mean modal-label ratio was 97.5%.

On its limited, imbalanced 16-case support (12 low, one medium, and three high), the LLM modal label achieved 75.0% accuracy, 32.0% Macro-F1, a 7.7% FSR, and a 33.3% under-escalation rate; these values are descriptive of the protocol stability audit only and should not be generalized. The large gap between accuracy and Macro-F1 reflects the skewed reference distribution in this selected protocol subset (12 low, one medium, and three high): most correct decisions were low-severity, whereas the rare medium and high-severity cases were poorly discriminated. The 75.0% accuracy should therefore not be interpreted as balanced assessment performance. These values are descriptive for the stability protocol only and are not compared as a head-to-head full-cohort result with the 130-case deterministic-rule evaluation.

## 5. Discussion

### 5.1. Sensing Support Is Part of the Assessment Evidence

This study treated sparse probe-vehicle sensing as a property of the measurement process rather than as a preprocessing inconvenience. The controlled-missingness results show why this distinction matters. Under high temporal-block missingness, GRU-Mask MAE remained close to the low-severity value, whereas the observed-target ratio fell to 12.1%. A prediction metric computed on a reduced support set does not, by itself, establish that the sensing system is equally informative. Reporting coverage and observed-target support alongside error is therefore necessary for a defensible assessment of sparse traffic sensing. The assessment results provide a related finding. The speed-plus-mask rule was the strongest overall deterministic baseline on the 130 adjudicated cases, while the addition of coverage reduced the false-severe rate and eliminated false-severe classifications among reference low-severity cases. This reduction came with lower overall discrimination, particularly in the low-coverage stratum. The under-escalation rate was unchanged at 4.3% across deterministic variants, so the observed false-severe-rate reduction neither establishes superior high-severity detection nor removes the need to review missed high-severity cases.

Consequently, coverage should not be interpreted as direct congestion evidence. Its appropriate role is to express the reliability of speed-based evidence and to support an explicit over-escalation-control–classification trade-off. The moderate inter-annotator agreement (κ=0.464) implies that the reference labels, especially for medium severity, carry non-negligible uncertainty; the 130 cases are an adjudicated human reference rather than an objective ground truth. Differences in FSR below the annotator disagreement rate—such as the 5.6% to 2.8% reduction—should therefore be interpreted as directional rather than definitive.

The learned-model comparison reinforces this multi-metric interpretation. Although the RBF-SVM increased mean grouped out-of-fold accuracy relative to speed plus mask, the paired 95% intervals for accuracy, Macro-F1, balanced accuracy, and FSR all included zero. Secondary analysis identified a higher RBF-SVM true-low-to-high error rate, an RBF-SVM disadvantage, and higher RBF-SVM low-class recall, an RBF-SVM advantage, both with intervals excluding zero; the implication of the difference therefore depends on the metric. Selecting a method solely by accuracy or Macro-F1 would ignore this safety-relevant, class-specific directionality; speed plus mask remains competitive, while the learned results provide internal evidence that the prospective feature set is informative.

### 5.2. Implications for Evidence Fusion and LLM Assistance

The current deterministic spatial fusion did not improve the primary assessment metrics. Both the spatial variant and the full deterministic fusion rule produced a 28.0% false-severe rate, compared with 5.6% for the speed-plus-mask baseline. This does not invalidate spatial context as a source of information; instead, it shows that simple fixed thresholds for neighboring-region support can over-escalate an alert when regional correlation, road connectivity, and temporal alignment are not jointly calibrated. The spatial rule is therefore best viewed as an uncalibrated baseline whose failure motivates calibrated, topology-aware spatial evidence rather than the abandonment of spatial context. In the present evidence, the speed-plus-mask rule is the appropriate primary rule baseline, while the coverage-aware rule is useful where reducing false-severe traffic-state alerts is prioritized.

The LLM protocol demonstrated format and repeatability stability: all 320 structured calls were schema-complete, no calls failed, and 15 of 16 cases had perfect modal-label repeatability. The runner records any failed case–repetition for later checkpoint/resume; no automatic retry was required in the reported run. This protocol evidence does not establish end-to-end operational stability or validated assessment superiority. The LLM should therefore be positioned as a structured explanation and coordination component whose output validity, cost, and repeatability can be audited, rather than as a replacement for the human-grounded 130-case assessment evaluation. Whether the LLM-generated explanations improve human review decisions remains to be validated in a prospective user study.

### 5.3. Robustness Across Missingness Mechanisms

The three missingness mechanisms provide different stress tests and should not be collapsed into a single “missing-data rate” narrative. Random removal distributes losses across the matrix and was associated with a monotonic GRU-Mask MAE increase from 11.584 to 12.932 km/h as severity increased. Segment-index-block removal retained comparatively higher observed-target support at all three severities and produced an intermediate error pattern. In contrast, temporal-block removal removed contiguous periods and yielded the smallest observed-target ratio at high severity. Its apparently stable MAE must therefore be interpreted conditionally: it characterizes the subset of targets that remained observable after a block outage, not the complete traffic state during the outage.

This distinction has a practical implication for reporting. A sparse-sensing model should report at least three quantities for every stress condition: the error on observed targets, the observed-target ratio, and a description of the missingness mechanism. Without the latter two, a lower MAE may be caused by a changed evaluation support rather than a more reliable traffic-state estimate. The present results consequently support coverage-aware evaluation as a reporting standard, even when the primary prediction model itself is unchanged.

The additional baseline matrix supports the same restrained interpretation. HistGB and historical average remain competitive on the fixed masks, whereas persistence and ridge regression degrade more strongly. Thus, the central experimental conclusion is about mask-consistent evaluation and support reporting, rather than a claim that a single model dominates every sparse-sensing condition.

### 5.4. Failure Modes and Operational Implications

The rule ablation identifies two qualitatively different failure modes. First, a large speed drop can be inflated by an unusually high preceding observation. When coverage is also weak and neighbors do not corroborate a deterioration, a speed-only escalation can create a false-severe traffic-state alert. The coverage-aware variant was designed to address this failure mode; its low-coverage FSR was 0.0%, but the accompanying decline in accuracy shows that conservative suppression also misses discriminative information.

Second, the current spatial rule can over-count weakly aligned neighbor signals. Three design properties contribute: the supportive-neighbor definition counts any slow or congested neighbor with correlation ≥ 0.03, a threshold that treats almost any weakly positive correlation as corroboration; neighbor evidence is aggregated without propagation-delay or temporal alignment; and support is counted without topology-aware weighting. Its FSR reached 33.3% in the low-coverage stratum and 32.3% in the medium-coverage stratum. A safe revision should require temporal alignment, topology-aware connectivity, and calibrated correlation support before a neighbor can escalate severity. Until such calibration is validated, the spatial rule should be treated as an analysis condition rather than an operational alerting policy.

For a future deployment study, the results motivate evaluating a two-mode response design. A standard mode could use speed plus temporal mask evidence to prioritize sensitivity, whereas a conservative mode could use coverage-aware suppression where false-severe traffic-state alerts have a high operational cost. Cases with very low coverage, conflicting evidence, or no corroborating neighbors should be marked as uncertain and routed for review rather than silently converted into a confident low- or high-severity traffic-state alert.

### 5.5. Operational Deployment and Data Governance

The workflow is designed around a 15 min regional monitoring cycle: regional speed and coverage are updated, a next-window speed is predicted, and the assessment rule returns both a traffic-state severity level and evidence fields. Its end-to-end latency, throughput, and operator usability have not been evaluated in a live deployment. The coverage measure should be exposed to operators as a sensing-quality indicator, not hidden in a model feature vector. The deterministic evidence record—predicted speed, recent speed change, coverage history, and supporting neighbors—allows an operator to trace why an alert was generated or downgraded.

The LLM component is most suitable after this deterministic evidence record has been created. Its role is to return a schema-valid explanation, identify missing or conflicting evidence, and coordinate a compact region-level summary. It should not receive hidden ground truth, should not create an incident narrative from external assumptions, and should retain the prompt, response schema, latency, and token audit for each call. The source traffic-speed data were provided by the Xuzhou Transportation Comprehensive Administrative Law Enforcement Detachment; therefore, the reproducibility package separates access-restricted source data from shareable derived metrics, masks, manifests, and de-identified audit outputs.

The selective-coverage analysis provides a transparent starting point for setting review capacity: the study reports retained-set metrics when 10–50% of lowest-coverage cases are routed to review, but does not prescribe a universal cutoff. A responsible authority should define the review capacity, retention period, access control, aggregation level, and response to over-escalated traffic-state alerts before operational use. As an illustrative operating point, routing the lowest-coverage 20% of cases to review under the speed-plus-mask rule yields 76.9% retained-set accuracy with 7.4% FSR on the retained 104 cases; routing the lowest-coverage 50% raises retained-set accuracy to 84.6% (n=65). Such points are illustrative operational trade-offs, not universal recommendations.

Table 9 consolidates the evidence-to-claim links used for deployment interpretation, including the supporting experiment, the numerical evidence, and the boundary that prevents each result from being generalized beyond the single-city, retrospective study.

### 5.6. Limitations and Future Work

Several limitations delimit the conclusions. First, the analysis uses one city and a three-month observation period (January–March 2026); transferability to other traffic networks, probe-vehicle penetration levels, and traffic patterns in summer, holidays, or other seasons remains unverified. The framework is transferable in structure, but the absolute speed thresholds and the coverage calibration are not demonstrated to be transferable across networks. Second, the controlled segment-index-block mechanism removes contiguous blocks of segment indices; because segment indices are not verified to be spatially contiguous, this is an index-based stress test rather than a demonstrated topology-aware outage, and it does not model every topology-aware sensor failure. Third, the deterministic thresholds were transparent design choices rather than learned or externally calibrated clinical-style decision thresholds. The present thresholds use absolute regional speeds and were not normalized by segment-specific free-flow speeds; their interpretation may therefore vary with road mix and should not be transferred unchanged to another network. Regional aggregation also improves observability but may mask intra-region heterogeneity (e.g., a congested arterial averaged together with free-flowing roads). Fourth, the adjudicated reference set is stratified and class-imbalanced, with only 17 medium-severity cases; it supports method comparison but is not a prevalence estimate for a deployed warning system. Finally, the LLM evaluation covers 16 selected cases from seven available initial-severity-by-coverage strata; the low-severity/low-coverage stratum was structurally unavailable under the initial-severity rule. Its reference distribution (12 low, one medium, and three high) differs from the full 130-case cohort (90 low, 17 medium, and 23 high), so its accuracy-related measures have wide uncertainty and cannot support population-level conclusions. In addition, the 32-region partition was constructed from the full three-month record (Section 3.1); the learned classifier comparison is an internal repeated grouped validation on the same 130 adjudicated cases and remains vulnerable to the small medium class, class imbalance, and single-city support; the common 243-configuration sensitivity audit contains duplicate listed settings for rules that do not use coverage or neighbor-count parameters; and post hoc exclusion of disputed or lower-confidence labels changed the apparent method ranking. These issues require external reference cohorts, nested model selection if future tuning is introduced, and prospective validation. The frozen 130-case evidence package also inherited full-period region correlations for ranking and summarizing neighbors. The post hoc train-only sensitivity audit changed no deterministic labels and only modestly shifted learned-comparator metrics, but it retained the frozen neighbor identities and order and therefore does not constitute a fully prospective reconstruction. The corrected implementation now estimates correlations from the training partition; future external validation should rebuild neighbor selection from training-only data before annotation.

The retained study artifacts do not provide stable vehicle identifiers, fleet-class composition, trip purpose, or an independently measured probe penetration rate. Accordingly, the sample cannot be claimed representative of a specific vehicle fleet, and the effect of fleet composition on regional coverage remains unmeasured.

Future work should expand the reference labels across cities and traffic conditions, use topology-aware outage simulations, calibrate traffic-state-severity thresholds on held-out data, and evaluate prospective alerts with real operational outcomes. A larger LLM protocol should retain fixed prompts and schema auditing while testing whether explanations improve human review, rather than only whether the model repeats its own labels. A natural next step is a prospective study that deploys the selective-review policy in a live monitoring dashboard and collects operator feedback on routed cases, including whether LLM-generated explanations improve review decisions.

## 6. Conclusions

This work developed and evaluated a mask- and coverage-aware framework for traffic-state assessment under sparse probe-vehicle sensing. The framework preserves regional speed, observation coverage, temporal history, and neighboring-region evidence as distinct inputs; evaluates prediction under random, temporal-block, and segment-index-block missingness; and compares fixed deterministic rules with learned assessment comparators against the same 130 adjudicated human labels. This design makes the sensing support visible throughout prediction, assessment, and evaluation rather than treating missing observations as an unreported preprocessing detail.

Three conclusions follow from the evidence. First, GRU-Mask outperformed the historical-average baseline across the nine controlled-missingness conditions, while HistGB remained a competitive non-neural reference with the lowest mean MAE (11.860 km/h); observed-target support changed substantially across mechanisms and severities, so prediction error must be interpreted together with evaluation support. Second, for human-grounded assessment, the RBF-SVM achieved the highest mean grouped out-of-fold accuracy (75.0%) and Macro-F1 (64.6%), whereas speed plus mask achieved 69.2% accuracy, 63.7% Macro-F1, higher balanced accuracy, and lower FSR. The paired intervals for the four headline metrics included zero. Secondary analysis found a higher RBF-SVM true-low-to-high error rate, an RBF-SVM disadvantage, and higher RBF-SVM low-class recall, an RBF-SVM advantage, with both intervals excluding zero; the cohort therefore shows metric-dependent differences rather than a uniformly superior assessment method. Adding coverage reduced FSR from 5.6% to 2.8% but lowered overall discrimination, and the common-grid sensitivity audit confirmed this trade-off across parameter settings. Label-uncertainty sensitivity also changed method rankings, so the adjudicated cohort should be treated as an internal human-grounded comparison rather than definitive ground truth. Third, the current spatial fusion thresholds over-escalated traffic-state alerts and require calibration, whereas the structured LLM protocol was highly repeatable and schema-valid on 16 cases but lacks support for a full-cohort effectiveness claim.

For prospective operational evaluation, the evidence motivates an auditable, reliability- aware workflow rather than an autonomous black-box warning system. A monitoring service could expose speed, coverage, temporal change, and neighbor evidence; choose a standard or conservative rule according to the cost of over-escalated traffic-state alerts; and route highly uncertain cases to review. Cross-city reference labeling, topology-aware outage experiments, calibrated spatial fusion, and prospective traffic-state alert evaluation are necessary before claims of general deployment readiness can be made.

## Figures and Tables

**Table 1:** Summary of related research streams and the positioning of this study under sparse sensing.

Research	Primary Objective	Sensing Support Retained	Reference and Audit Evaluation
Probe-based state estimation	Estimate speed, density, and state from mobile observations	Sometimes	Estimation error; human references and LLM audits are uncommon
Traffic imputation and forecasting	Impute unavailable values or reduce forecast error	Sometimes	Value errors; matched support and review-policy effects are uncommon
Traffic-state alerting and decision support	Identify or communicate operational traffic states	Inconsistent	Operational labels may be used; separate explanation and review evaluation is rare
This paper	Assess ordinal traffic-state severity while preserving sensing reliability	Yes: mask, coverage, and zeros	Matched baselines; 130 adjudicated cases; threshold robustness; selective review; separate 16-case audit

**Table 2:** Terminology, abbreviations, and notation. Each abbreviation is expanded at its first occurrence in the main text.

Term or Symbol	Definition	Unit/Range
Probe-vehicle sensing (PVS)	Mobile observations contributed by probe vehicles and mapped to road segments.	-
$R_{t,r}$	Regional speed-matrix entry $R_{t,r}$: probe-count-weighted mean of observed analysis-segment speeds in region r at time t; missing when $M_{t,r}=0$.	km/h
$C_{t,r}$	Coverage-matrix entry $C_{t,r}$: observed analysis segments divided by all historically observable analysis segments in region r; the denominator is fixed over time.	[0, 1]
M	Observation-mask entry $M_{t,r}$: 1 if at least one analysis segment in region r is observed at time t, and 0 otherwise.	{0, 1}
${Corr}_{r,q}$	Historical Pearson correlation between regional speed series for regions r and q.	[−1, 1]
Gated recurrent unit with mask input (GRU-Mask)	Recurrent predictor using regional speed and coverage channels.	-
Mean absolute error (MAE)	Mean absolute prediction error evaluated only on observed targets.	km/h
Macro F1-score (Macro-F1)	Unweighted mean of the F1-scores for low, medium, and high severity.	[0, 1]
False-severe rate (FSR)	Fraction of non-high reference labels predicted as high	[0, 1]

**Table 3:** Information sets and temporal controls for a target region–time case. The target time is t+1; “available” means supplied to the stated component.

Field Group	Time Index and Source	Availability and Control
Target and neighbor speed/coverage histories	t−11 through t; observed regional histories and coverage.	Available to the predictor, deterministic rules, LLM, and annotators. Missing input speeds are handled as described in Section 3.4.
Target and neighbor predicted speeds	One-step predictions for t+1 generated from the t−11:t inputs.	Available to deterministic rules, LLM, and annotators. They are model outputs, not observed target-time measurements.
Neighbor correlation and adjacency summaries	Frozen 130-case package: correlation matrix estimated from the complete three-month record; revised implementation: chronological 70% training partition. Topology was fixed before case evaluation.	Available to deterministic rules, LLM, and annotators. No observed neighbor speed at t+1 is included in these summaries.
Target true speed and target-observation	Observed target value at t+1, if present, and its observation indicator.	Available only to annotators for offline reference labeling and adjudication; excluded from deterministic-rule decisions and removed before every LLM call.

**Table 4:** Main experimental and implementation configuration.

Item	Configuration
Study data	Xuzhou probe-vehicle observations; 1 January–31 March 2026; 15 min windows; 49,645 segments; 32 regions.
Prediction samples	Lookback = 12 windows; horizon = 1 window; chronological train/validation/test split = 6036/863/1729 samples.
Controlled missingness	Random, temporal-block, and segment-index-block mechanisms; nominal removal rates of 10%, 30%, and 50%.
GRU-Mask	Hidden size 96; dropout 0.1; learning rate 10^−3^; batch size 512; maximum 120 epochs; patience 10; seeds 42, 123, and 2026.
Deterministic assessment	Five evidence-fusion variants; three ordinal labels; no human labels used to construct rules.
Human reference set	130 cases; two independent annotators; disagreement adjudication.
LLM stability protocol	DeepSeek V4 Flash (API alias: deepseek-chat); 16 cases; 5 repetitions per case; temperature 0; four structured stages per repetition.
Uncertainty analysis	Original stratified analyses: 1000 case-level bootstrap resamples, seed 42; expanded same-cohort comparison: 2000 paired sample_index-cluster bootstrap resamples, seed 20260920.
Assessment comparators	12 prospective features; balanced logistic regression and RBF-SVM; 25 repeats of four-fold grouped validation by sample_index; no tuning.

**Table 5:** Prediction performance under controlled missingness. GRU-Mask values are mean ± standard deviation over three seeds; the observed-target ratio denotes the evaluable fraction of test targets.

Mechanism	Severity	Removal (%)	Observed Targets (n)	Observed Targets (%)	GRU-Mask MAE	GRU-Mask RMSE
Random	Low	10	13,187	23.8	11.584 ± 0.010	15.883 ± 0.012
	Medium	30	12,210	22.1	12.149 ± 0.017	16.508 ± 0.017
	High	50	10,860	19.6	12.932 ± 0.016	17.310 ± 0.024
Temporal block	Low	10	12,055	21.8	11.269 ± 0.003	15.553 ± 0.002
	Medium	30	9535	17.2	11.347 ± 0.002	15.600 ± 0.016
	High	50	6719	12.1	11.276 ± 0.009	15.480 ± 0.017
Segment-index block	Low	10	13,230	23.9	11.580 ± 0.011	15.916 ± 0.011
	Medium	30	12,423	22.5	11.718 ± 0.015	16.093 ± 0.022
	High	50	11,343	20.5	12.020 ± 0.015	16.434 ± 0.011

**Table 6:** Independent annotation reliability before adjudication.

Paired Cases	Direct Agreement (%)	Cohen’s κ	Disagreements
130	66.2	0.464	44

**Table 7:** Traffic-state assessment performance on 130 adjudicated human labels. Deterministic rules are fixed full-cohort evaluations; logistic regression and RBF-SVM values are means over 25 repetitions of four-fold grouped out-of-fold validation. FSR is the false-severe rate among non-high reference labels. Values are percentages with paired sample_index-cluster-bootstrap 95% confidence intervals; no model was selected or tuned on this cohort.

Variant	Accuracy (%)	Macro-F1(%)	FSR (Non-High→High) (%)	False-Severe Rate Among True Low-Severity Labels(%)	Under-Escalation (%)	Balanced Accuracy (%)	Mean Ordinal Distance
Speed only	68.5 [59.8, 76.4]	62.8 [53.4, 71.1]	4.7 [1.0, 8.9]	1.1 [0.0, 3.6]	4.3 [0.0, 15.0]	68.2 [57.3, 78.6]	0.331 [0.246, 0.425]
Speed + mask	69.2 [60.9, 77.3]	63.7 [54.2, 72.2]	5.6 [1.9, 10.2]	2.2 [0.0, 5.9]	4.3 [0.0, 15.0]	69.7 [58.5, 79.9]	0.331 [0.242, 0.427]
Speed + mask + coverage	58.5 [50.0, 66.4]	57.5 [48.6, 65.5]	2.8 [0.0, 6.4]	0.0 [0.0, 0.0]	4.3 [0.0, 15.0]	63.9 [53.4, 73.6]	0.423 [0.336, 0.512]
Speed + mask + coverage + spatial	54.6 [46.2, 62.8]	46.0 [37.9, 53.8]	28.0 [19.4, 37.6]	18.9 [11.3, 27.9]	4.3 [0.0, 15.0]	54.8 [46.9, 63.3]	0.592 [0.472, 0.726]
Full deterministic fusion rule	54.6 [46.2, 62.8]	46.0 [37.9, 53.8]	28.0 [19.4, 37.6]	18.9 [11.3, 27.9]	4.3 [0.0, 15.0]	54.8 [46.9, 63.3]	0.592 [0.472, 0.726]
Majority-class (always low)	69.2 [61.1, 77.0]	27.3 [25.3, 29.0]	0.0 [0.0, 0.0]	0.0 [0.0, 0.0]	100.0 [100.0, 100.0]	33.3 [33.3, 33.3]	0.485 [0.356, 0.619]
Balanced logistic regression (grouped OOF)	74.2 [66.9, 81.1]	62.8 [53.3, 71.0]	9.1 [4.3, 14.6]	5.6 [2.0, 9.8]	7.0 [0.6, 17.7]	66.1 [56.2, 75.4]	0.309 [0.222, 0.402]
RBF-SVM (grouped OOF)	75.0 [67.6, 81.6]	64.6 [55.3, 72.7]	9.7 [4.7, 15.5]	7.6 [3.2, 12.9]	6.1 [0.2, 16.7]	68.5 [58.7, 77.4]	0.313 [0.222, 0.412]

**Table 8:** LLM protocol stability and limited-sample assessment evaluation. Classification metrics use only the 16 cases in the stability protocol; the low-severity/low-coverage stratum was structurally unavailable, and the 16-case metrics are descriptive of protocol stability only (Section 4.9).

**Cases**	**Repetitions**	**Calls**	**Schema (%)**	**Modal-Label Ratio (%)**	**Perfect Cases**
16	5	320	100	97.5	15/16
**Classification metrics**	**Accuracy (%)**	**Macro-F1 (%)**	**FSR (%)**	**Under-escalation (%)**	
16-case modal label	75.0	32.0	7.7	33.3	

**Table 9:** Evidence-to-claim map for the main conclusions and their boundaries.

Evidence	Supported Claim	Boundary of Interpretation
Nine controlled-missingness conditions with three GRU seeds	Prediction error and evaluation support vary jointly with missingness mechanism and severity.	One city and three months; only observed targets contribute to MAE and RMSE.
130 adjudicated cases	Speed + mask is the strongest overall deterministic baseline in this cohort.	Stratified reference set; not a prevalence estimate or external validation.
Coverage-stratified assessment metrics	Coverage-aware rules can reduce false-severe traffic-state alerts under weak sensing.	Lower FSR is accompanied by lower discrimination, particularly at low coverage.
Spatial-rule ablation	The present spatial thresholds over-escalate traffic-state alerts.	This does not establish that spatial information is unhelpful after recalibration.
16-case LLM protocol, 320 calls	Fixed prompts and schemas produced repeatable, valid structured outputs.	Stability evidence is not full-cohort assessment-effectiveness evidence.

## Data Availability

The traffic speed data used in this study were provided by the Xuzhou Transportation Comprehensive Administrative Law Enforcement Detachment and are access restricted. OpenStreetMap open-source road-network data were used for map matching. The derived experimental results, evaluation masks, and support-chain audit outputs generated in this study are available from the corresponding author upon reasonable request.

## Appendix A. Software, Hardware, and Reproducibility

All experiments were executed through versioned scripts that produce a manifest together with each derived dataset, prediction output, annotation artifact, and evaluation table. Table A1 records the archival environment used for the project. The final GRU-Mask profiles identify the Apple Metal Performance Shaders (MPS) device; the environment record below is retained to enable reproduction of the pipeline and regeneration of the reported tables and figures.

**Table A1:** Software and hardware environment recorded for reproducibility.

Item	Recorded Configuration
Operating system	macOS 26.5.2 (arm64)
Hardware	MacBook Pro (Mac16,7), Apple M4 Pro, 14 CPU cores (10 performance and four efficiency cores), 24 GB unified memory
Accelerator	Apple Silicon Metal Performance Shaders (MPS) for final GRU-Mask runs; no discrete GPU was used
Python	Python 3.11.15 in the Conda environment (https://docs.conda.io/en/latest/, accessed on 1 October 2026)
Core packages	Primary prediction runs: NumPy 2.4.6; pandas 3.0.3; scikit-learn 1.9.0; PyTorch 2.14.0. dev20260706 (Meta Platforms, Inc., Menlo Park, CA, USA); Matplotlib 3.11.0. Same-cohort assessment and correlation-sensitivity runs: Python 3.12; NumPy 2.4.1; scikit-learn 1.8.0
Controlled missingness	Random seed 20260713; random, temporal-block, and segment-index-block mechanisms; 10%, 30%, and 50% nominal removal rates
GRU-Mask	Seeds 42, 123, and 2026; hidden size 96; dropout 0.1; learning rate 10^−3^; batch size 512; maximum 120 epochs; patience 10
Assessment uncertainty	1000 bootstrap resamples; seed 42
LLM protocol	DeepSeek V4 Flash (API alias: deepseek-chat) via https://api.deepseek.com; maximum 1200 response tokens; 90 s timeout; 16 cases; five repetitions per case; four structured calls per repetition.

For software provenance, GRU-Mask was implemented using PyTorch (Meta Platforms, Inc., Menlo Park, CA, USA) and trained using Apple MPS (Apple Inc., Cupertino, CA, USA). Data processing and statistical analysis used Python 3.11.15 (Python Software Foundation, Wilmington, DE, USA), together with the package versions listed in Table A1. The primary non-learning prediction baselines—historical average, persistence, moving average, and ridge regression—were evaluated on CPU and do not require learning-rate, batch-size, or epoch settings. The LLM protocol used the DeepSeek V4 Flash model (API alias: deepseek-chat) through the DeepSeek API with a fixed temperature of 0. The run manifest records the provider, endpoint, model alias, execution timestamp, token limit, timeout, prompt/schema identifiers, and checkpoint/resume behavior; request traces, schema audits, and token counts are retained in the project audit records and are available from the corresponding author upon reasonable request.

The executable sequence is: generate controlled-missingness matrices; derive regional features; construct chronological prediction datasets; train prediction models; generate rule predictions; validate and adjudicate annotations; run the limited LLM protocol; and calculate final assessment metrics. The retained run manifests identify the input paths, output paths, configuration, seeds, and the status of every completed stage. The data-availability statement specifies the access restriction on the source traffic-speed data; derived outputs are available from the corresponding author upon reasonable request.

For artifact-level reproducibility, the frozen manifests and directory names retain the legacy identifier spatial_block for the segment-index-block condition. The identifier was not renamed because doing so would break direct traceability to the executed outputs; it does not denote a topology- or geometry-based outage.

## Appendix B. Human Annotation and Adjudication Protocol

Each reference case was a region–time record containing the predicted speed, latest observed speed, speed history, target-observation status, coverage history, sparsity warning, and neighboring-region summary. Two annotators independently selected one ordinal operational traffic-state label (low, medium, or high), a confidence score from 1 to 4, an uncertainty flag, any affected neighboring regions, and an evidence note. Table A2 summarizes the qualitative decision aids supplied to both annotators; throughout annotation, low coverage indicated limited reliability rather than congestion by itself. In this description, predicted speed denotes the GRU one-step output shown as case evidence; the annotators remained blind to deterministic assessment labels, LLM assessment outputs, and all pre-label behavior analyses.

**Table A2:** Human annotation decision aids. These are qualitative guidance rules and were not applied as automatic label thresholds.

Label or Field	Guidance Provided to Annotators
Low severity	No material low-speed, deterioration, or neighbor-propagation evidence; sparse coverage alone is insufficient for escalation.
Medium severity	Borderline low speed, a material decline under sparse support, or partially consistent neighboring evidence.
High severity	Consistent evidence of severe low speed, persistent deterioration, and/or multiple supporting neighboring regions.
Sparse-data uncertainty	Marked when coverage is very low, zero-coverage history is present, the target is unobserved, or evidence conflicts.
Confidence	Integer from 1 (very uncertain) to 4 (certain), reflecting the strength and consistency of the available evidence.

The low label was used when available speed, temporal, and neighbor evidence did not support a material traffic-state alert. The medium label captured a borderline low-speed signal, a speed decline under sparse support, or partially consistent neighbor evidence. The high label required consistent evidence of severe low speed, a persistent deterioration, and/or multiple supporting neighboring regions. A large apparent speed drop following an unusually high previous speed was treated cautiously, particularly when coverage was low. These rules were guidance for structured human judgment, not automatic label thresholds.

The two annotation files were completed independently before agreement was calculated. The rule outputs, LLM outputs, and all pre-label behavior analyses were not supplied to annotators. Direct agreement and Cohen’s kappa were then calculated for all complete pairs. Only disagreements were reviewed during adjudication, and the final adjudicated label was recorded for every case. The resulting reference set contains 130 cases; it combines the initial 120-case package with 10 supplementary cases selected to improve coverage of available strata. The initial low-severity/low-coverage stratum was structurally unavailable under the original initial-severity rule and is reported as a design limitation rather than silently treated as a sampling failure.

### Annotator Contribution Statement

Hongxin Wang and Zhicheng Shen had each completed a bachelor’s degree in transportation and were pursuing master’s study in the same discipline at the time of annotation. Their academic preparation included traffic-flow theory, traffic engineering, traffic-state identification, and transportation-data analysis. Task-specific preparation consisted of jointly reviewing the written annotation protocol and the decision aids in Table A2. No pilot labeling, example walkthrough, calibration exercise, or discussion of any formal study case took place before independent annotation. They then completed the two annotation passes independently and provided adjudication support for disagreement records. They did not receive deterministic-rule outputs, LLM outputs, or pre-label behavior analyses. Their contribution was limited to the human-annotation and adjudication workflow; they did not design the assessment rules, train the prediction models, or conduct the LLM evaluation.

## Appendix C. Audit and Reproducibility Records

Detailed audit outputs are retained in the project records. They include 1000-resample bootstrap confidence intervals; coverage-, source-package-, and initial-severity-stratified classification metrics; confusion matrices; the label-free 243-configuration threshold analysis; post-label robustness metrics for the same prespecified grid; coverage-selective classification metrics; and case-level LLM repeatability, schema validation, latency, and token-use records. The annotation validation summary, final adjudication file, per-case assessment prediction join, coverage profiles, dataset manifests, and machine-readable figure data are available from the corresponding author upon reasonable request. These records retain the separation between (i) human-grounded evaluation, (ii) label-free rule and LLM behavior analyses, and (iii) LLM protocol-stability evidence, preventing post hoc use of assessment-system outputs to redefine the human reference labels.

### Appendix C.1. LLM Prompt and Schema Summary

The LLM protocol uses a fixed system-level constraint: “You are a traffic-state assessment agent for sparse probe-vehicle data. You must reason from the given JSON only. Do not invent incidents, weather, or external events. Return valid compact JSON only.” The case input contains only the prediction, historical speed and coverage, region metadata, and neighboring evidence. Target true speed and target-observation outcome are removed before every LLM call. Consequently, the LLM cannot directly access the variable used to judge prediction support or the human reference label.

Table A3 summarizes the four fixed prompt stages and their required structured outputs.

**Table A3:** Four-stage LLM prompt and schema summary. All stages used fixed prompts, temperature 0, and JSON schema validation.

Stage	Prompt Focus	Required Structured Output
DataQuality Agent	Assess coverage, zero-coverage history, missingness pattern, and reliability limitations.	Data reliability; sparsity warning; missing pattern; warnings; suggested handling; reason.
Region Agent	Assess target-region traffic state from predicted speed, recent history, and reliability context.	State; traffic-state severity level; confidence; evidence-consistent hypotheses; neighboring regions to check; reason.
Neighbor-Evidence Agent	Assess whether neighboring regions provide contemporaneous evidence consistent with the target-region state. Its name does not imply a validated forecast of propagation direction, range, or timing.	Neighbor-evidence severity; affected regions to review; neighbor evidence; uncertainty note; reason.
Coordinator	Integrate the three preceding JSON outputs without adding external context.	Global traffic-state severity level; top-severity regions; uncertainty notes; concise explanation.

For each selected case, the four stages were run five times. A schema audit checked that every required field was present and used an allowed value. The final LLM label was the modal coordinator severity label across repetitions. Full prompt templates, JSON schemas, planned calls, and de-identified trace audits are retained in the project audit records and are available from the corresponding author upon reasonable request rather than reproduced verbatim in the main manuscript.

### Appendix C.2. Additional Sensing-Support and Class-Specific Results

Table A4 summarizes the distribution of observed analysis segments and valid matched records across the complete set of region–time cells before controlled masking. Positive-support cells are reported separately because the full distribution contains a substantial point mass at zero.

**Table A4:** Distribution of observed segments and valid matched records across 276,480 region–time cells before controlled masking.

Measure	Population	Cells (n)	Zero (%)	Median [IQR]	P90/P95	Maximum
Observed segments	All region–time cells	276,480	43.9	1 [0–3]	7/10	45
Observed segments	Positive-support cells	155,071	0.0	3 [1–6]	10/13	45
Valid matched records	All region–time cells	276,480	43.9	1 [0–3]	7/11	53
Valid matched records	Positive-support cells	155,071	0.0	3 [1–6]	10/14	53

Note: Valid matched records are observations contributing to aggregation after application of the 0–140 km/h speed filter; they are not unique vehicles. Of 685,805 successfully map-matched records, one 143 km/h record was excluded, leaving 685,804 records in the aggregation matrices.

**Table A5:** Class-specific assessment performance for four principal comparisons. Deterministic rules use fixed full-cohort predictions; majority and RBF-SVM values are means across 25 grouped out-of-fold repetitions.

Method	Evaluation	Class	Precision	Recall	F1-Score
Majority baseline	25-repeat grouped OOF mean	Low	0.692	1.000	0.818
Majority baseline	25-repeat grouped OOF mean	Medium	n/a	0.000	0.000
Majority baseline	25-repeat grouped OOF mean	High	n/a	0.000	0.000
Speed + mask	Fixed full cohort	Low	0.969	0.689	0.805
Speed + mask	Fixed full cohort	Medium	0.273	0.706	0.393
Speed + mask	Fixed full cohort	High	0.727	0.696	0.711
Coverage-aware	Fixed full cohort	Low	0.961	0.544	0.695
Coverage-aware	Fixed full cohort	Medium	0.210	0.765	0.329
Coverage-aware	Fixed full cohort	High	0.824	0.609	0.700
RBF-SVM	25-repeat grouped OOF mean	Low	0.943	0.806	0.869
RBF-SVM	25-repeat grouped OOF mean	Medium	0.373	0.616	0.464
RBF-SVM	25-repeat grouped OOF mean	High	0.585	0.631	0.606

Note: Precision is reported as n/a when a baseline makes no prediction in that class. The corresponding recall and F1-score are reported as 0.000. The RBF-SVM statistics are calculated within each grouped out-of-fold repetition and then averaged, reproducing the reported mean Macro-F1 of 64.6%.
